# Association Between Sleep Duration and Cognitive Decline

**DOI:** 10.1001/jamanetworkopen.2020.13573

**Published:** 2020-09-21

**Authors:** Yanjun Ma, Lirong Liang, Fanfan Zheng, Le Shi, Baoliang Zhong, Wuxiang Xie

**Affiliations:** 1 Peking University Clinical Research Institute, Peking University First Hospital, Beijing, China; 2 Clinical Epidemiology Department, Beijing Chaoyang Hospital, Capital Medical University, Beijing, China; 3 Brainnetome Center, Institute of Automation, Chinese Academy of Sciences, Beijing; 4 National Clinical Research Center for Mental Disorders, Key Laboratory of Mental Health and Peking University Sixth Hospital, Peking University, Beijing, China; 5 Department of Geriatric Psychiatry, Affiliated Wuhan Mental Health Center, Tongji Medical College of Huazhong University of Science and Technology, Wuhan, China

## Abstract

**Importance:**

An association between sleep duration and the trajectory of cognitive decline has not been conclusively demonstrated.

**Objective:**

To investigate the association between sleep duration and cognitive decline by a pooled analysis of 2 nationally representative aging cohorts.

**Design, Setting, and Participants:**

A pooled cohort study using data from waves 4 to 8 (2008-2009 to 2016-2017) in the English Longitudinal Study of Ageing and waves 1 to 3 (2011 to 2015) in the China Health and Retirement Longitudinal Study in a population-based setting. Participants were 2 randomly enrolled cohorts comprising 28 756 individuals living in England who were 50 years or older and those living in China who were 45 years or older.

**Exposure:**

Self-reported sleep duration per night according to face-to-face interviews.

**Main Outcomes and Measures:**

Global cognitive *z* scores were calculated according to immediate and delayed recall test, an animal fluency test, the serial sevens test, an intersecting pentagon copying test, and a date orientation test.

**Results:**

Data were analyzed from 20 065 participants, including 9254 from the English Longitudinal Study of Ageing (mean [SD] age, 64.6 [9.8] years; 55.9% [5174 of 9254] women; median follow-up duration, 8 [interquartile range, 6-8] years) and 10 811 from the China Health and Retirement Longitudinal Study (mean [SD] age, 57.8 [9.0] years; 50.2% [5425 of 10 811] men; median follow-up duration, 4 [interquartile range, 4-4] years). During 100 000 person-years of follow-up, global cognitive *z* scores in individuals with 4 hours or less (pooled β = −0.022; 95% CI, −0.035 to −0.009 SD per year; *P* = .001) and 10 hours or more (pooled β = −0.033; 95% CI, −0.054 to −0.011 SD per year; *P* = .003) of sleep per night declined faster than in the reference group (7 hours per night) after adjusting for a number of covariates. An inverted U-shaped association between sleep duration and global cognitive decline was also observed.

**Conclusions and Relevance:**

In this pooled cohort study, an inverted U-shaped association between sleep duration and global cognitive decline was found, indicating that cognitive function should be monitored in individuals with insufficient (≤4 hours per night) or excessive (≥10 hours per night) sleep duration. Future studies are needed to examine the mechanisms of the association between sleep duration and cognitive decline.

## Key Points

**Question:**

What is the association between sleep duration and cognitive decline in the general aging population?

**Findings:**

During 100 000 person-years of follow-up in this pooled cohort study of 28 756 individuals, global cognitive function in individuals with extreme sleep duration (≤4 or ≥10 hours per night) declined statistically significantly faster than in the reference group (7 hours per night) after adjusting for covariates. An inverted U-shaped association between sleep duration and global cognitive decline was also observed.

**Meaning:**

The inverted U-shaped association indicates that cognitive function should be monitored in individuals with insufficient or excessive sleep duration.

## Teaser

This pooled cohort study investigates the association between sleep duration and cognitive decline in a combined analysis of 2 nationally representative aging cohorts.

## Introduction

The proportion of older people has increased rapidly in recent decades such that 12% of the global population in 2015 was 60 years or older.^1^ By 2050, this proportion is projected to be greater than one-fifth of the population.^1^ Consequently, the number of older adults with cognitive impairment and dementia is increasing rapidly.^2^ Dementia is one of the most common and serious disorders in later life. It is responsible for a large proportion of disability and mortality in older people and imposes a huge burden of long-term care for families and society.^3^ No effective therapies are available for treating dementia; therefore, the development of dementia prevention strategies has become a priority.^4,5^ During the long preclinical phase of dementia, accelerated cognitive decline is regarded as a cardinal marker. Thus, the identification of risk factors for cognitive decline is of great significance for dementia prevention.

Previous studies have reported a strong association between sleep and cognitive function in older adults.^6^ Although a number of cohort studies^7,8,9,10,11,12,13,14,15,16,17,18,19,20,21,22,23,24^ have examined whether baseline sleep duration was associated with cognitive decline and incident dementia, the results were inconsistent. Some cohort studies^7,8,9,10,11^ observed sleep duration to be statistically significantly associated with cognitive decline and incident dementia, whereas other studies^12,13,14,15,16,17,18,19,20,21,22,23,24^ did not reach this conclusion. However, the sample sizes of most previous studies^11,12,14,15,16,18,19,20,21,22,24^ were small. In addition, the magnitude of the association between sleep duration, especially extreme sleep duration (≤4 or ≥10 hours per night), and various cognitive domains is unclear. Therefore, a large cohort study is needed to explore the association between sleep duration and the trajectory of cognitive decline.

The English Longitudinal Study of Ageing (ELSA)^25^ and the China Health and Retirement Longitudinal Study (CHARLS)^26^ are both nationally representative studies of aging cohorts with multiwave data sets, including cognitive assessments and large sample sizes. These studies enabled investigation of the longitudinal association between baseline sleep duration and consequent cognitive decline. Hence, in the present study, we sought to assess the association between sleep duration and the trajectory of subsequent cognitive decline by pooling and analyzing data from these 2 cohorts.

## Methods

### Study Population

We affirm that all procedures contributing to this pooled cohort study comply with the ethical standards of the relevant national and institutional committees on human experimentation and with the Declaration of Helsinki,^27^ as revised in 2013. The data were derived from 2 community-based nationally representative longitudinal cohort studies conducted in the United Kingdom and China (waves 4-8 [2008-2009 to 2016-2017] in the ELSA and waves 1 to 3 [2011 to 2015] in the CHARLS) in a population-based setting, which were approved by the London Multicenter Research Ethics Committee and the Peking University Institutional Review Board, respectively. All participants in the 2 cohorts provided written informed consent. Deidentified data from the 2 cohorts were used in this pooled analysis. This study followed the Strengthening the Reporting of Observational Studies in Epidemiology (STROBE) reporting guideline.

eFigure 1 in the Supplement shows flowcharts describing the participant selection process for this study. The ELSA sample comprised 11 050 individuals 50 years or older who were participants in the Health Survey for England, which randomly enrolled individuals living in England based on postcode.^25,28,29^ The ELSA commenced in 2002-2003 (wave 1), and follow-up assessments were conducted every 2 years until 2016-2017 (wave 8). Because sleep duration was first included in the assessment during wave 4 of the ELSA, wave 4 (2008-2009) was regarded as the baseline of the present study, and we assessed data from the follow-up assessments conducted during waves 5 to 8. The CHARLS cohort included 17 706 randomly selected Chinese residents 45 years or older who were enrolled via multistage probability sampling.^26^ The first wave of data collection in the CHARLS took place in 2011, and follow-up surveys were conducted at 2-year intervals until 2015. Because the participant sleep duration data were collected during the first wave, data from wave 1 (2011 [baseline]) to wave 3 (2015) of the CHARLS were used in the present study. This study included data from 20 065 participants, 9254 from the ELSA and 10 811 from the CHARLS, for whom the baseline data sets were complete and at least 1 remeasurement of cognitive functioning had been conducted (eFigure 1 in the Supplement).

### Sleep Duration

Baseline sleep duration was self-reported without any given categories in face-to-face interviews. Participants were asked to report the “number of hours of sleep on an average week night” (in the ELSA) and to respond to the question “During the past month, how many hours of actual sleep did you get at night (average hours for one night)?” (in the CHARLS). The participants were divided into 7 groups according to sleep duration (≤4, 5, 6, 7, 8, 9, or ≥10 hours per night) for the analyses (eMethods in the Supplement).

### Cognitive Assessment

Cognitive assessment was conducted in all waves and included the 3 aspects of memory, executive function, and orientation. The memory and orientation function tests were the same for the 2 cohorts, whereas the executive function assessment differed.

The memory assessment task comprised immediate and delayed word recall for 10 unrelated words. The memory score was the sum of words that were successfully recollected in the immediate and delayed word recall tasks, separately, and ranged from 0 to 20. The orientation test comprised 4 questions regarding the day of the week, the month, the date of the month, and the year. One point was given for each correct answer. In the ELSA, individuals were asked to name as many animals as they could in 1 minute, and the number of animal names was counted as the executive score. In the CHARLS, executive function was assessed using the serial sevens test, in which the participant counts backward from 100 in increments of 7 (5 successive counts, with 1 point given for each correct answer), and by copying intersecting pentagons, in which the participant is asked to observe and draw a picture of 2 overlapping pentagons (3 points were given for a successful drawing and 0 points for an unsuccessful drawing). The executive score was the sum of these 2 tests and ranged from 0 to 8. Both the reliability and the validity of these tests have been well documented^30,31,32,33^ (eMethods in the Supplement).

The *z* Scores of the cognitive function test data were generated to compare across tests. Specifically, each test score was standardized according to the baseline score by subtracting the mean and dividing by the SD. For example, given a baseline mean memory score of 10.6889994 and SD of 3.4863824 in the ELSA, a memory score of 12 points at any wave in the ELSA would be converted into a standardized memory *z* score as follows: (12 − 10.6889994) / 3.4863824. Therefore, an individual with original memory scores of 12, 11, 8, 9, and 9 at waves 4 to 8 in the ELSA had corresponding *z* scores of 0.38, 0.09, −0.77, −0.48, and −0.48, respectively. The *z* score for global cognitive function was calculated by averaging the *z* scores for the 3 tests and restandardizing to baseline according to the mean and SD of the baseline global cognitive *z* scores. This approach has been widely adopted to calculate *z* scores of global cognitive function.^30,31,32,33,34^

### Covariates

Covariates that might confound the association between sleep duration and cognitive function in the analyses included sex, age (in years), body mass index (calculated as weight in kilograms divided by height in meters squared), systolic blood pressure (in mm Hg), level of education, Center for Epidemiologic Studies Depression (CES-D) Scale score, cohabitation status, current smoking, alcohol consumption, diabetes, and self-reported history of coronary heart disease, stroke, cancer, chronic lung disease, or asthma. In the CHARLS, a high level of education was defined as completion of at least senior level of high school; in the ELSA, it was defined as at least the level 3 National Vocational Qualification or the General Certificate of Education Advanced Level, which is equivalent to senior high school. Cohabitation status indicated whether or not the participant currently lived alone. The ELSA used the 8-item version of the CES-D Scale to measure depressive symptoms.^35,36^ Each item was scored with a 0 or 1, with a maximum score of 8. As in previous studies,^36,37^ participants scoring 4 or more were defined as having depressive symptoms. At the CHARLS baseline, a 10-item version of the CES-D was used to assess depressive symptoms.^38^ Each item was scored from 0 (rarely or none of the time) to 3 (most or all of the time), for a summed score ranging from 0 to 30. According to prior validation studies,^38,39^ a score of 12 or higher was used to define depressive symptoms. Participants were divided into 2 groups of nonsmokers (including ex-smokers) and current smokers. Alcohol consumption was defined as drinking at least once per week over the previous year. Hypertension was defined as systolic blood pressure of at least 140 mm Hg, diastolic blood pressure of at least 90 mm Hg, and/or self-reported treatment of hypertension with antihypertensive medication. Diabetes was defined as self-reported physician-diagnosed diabetes or current use of antidiabetic therapy. Chronic disease measures included self-reported physician-diagnosed cancer, chronic lung disease, and asthma (eMethods in the Supplement).

### Statistical Analysis

The results are presented as number (percentage) for discrete variables and as mean (SD) or median (interquartile range [IQR]) for continuous variables. First, data were analyzed at the cohort level according to the following uniform protocol. In cross-sectional analyses, analysis of covariance was used to examine the association between hours of sleep per night and cognitive function at baseline after adjusting for the covariates mentioned previously. The results are presented as least-squares means with 95% CIs of cognitive *z* scores compared with the reference group (7 hours per night).

The multiple measures of cognitive function in individual participants constituted repeated-measures data. Linear mixed models were used to evaluate the longitudinal association between baseline sleep duration and consequent cognitive decline (SD per year) during follow-up, incorporate all available follow-up data on cognitive function, account for the correlations between repeated measures conducted in individual participants, and handle missing data. In these models, personal identification numbers were used to identify repeated measures of cognitive function, and both the slope and the intercept were fitted as random effects to account for different rates of cognitive decline during the follow-up assessments and interindividual differences at baseline, respectively. Models included baseline sleep duration per night, time (in years since baseline), baseline sleep duration by time interaction, and the covariates mentioned above. The regression coefficient of the interaction term indicated a differential change in cognitive *z* scores (SD per year) compared with the reference group (7 hours per night).

Pooled analyses were then conducted using random-effects meta-analyses, which take study heterogeneity into account, to generate pooled effect estimates and 95% CIs. The extent of variability across the studies that was attributable to heterogeneity beyond sampling error was estimated using the *I*^2^ statistic.

Both cross-sectional and longitudinal analyses were stratified by sex as sensitivity analyses. Statistical analyses were 2-sided, with α** = **.05 as the threshold for statistical significance, and were performed using SAS version 9.4 (SAS Institute Inc) and Stata version 11 (StataCorp LLC).

### Data Availability

The data sets from the ELSA and the CHARLS are freely available to all bonafide researchers. The UK Data Service and the CHARLS research team may be contacted to gain access to the ELSA^40^ and CHARLS^41^ data, respectively. The data can also be obtained on request from the corresponding author.

## Results

### Baseline Characteristics and Sample Size

Of 28 756 individuals who participated in the baseline survey, 20 065 had a complete baseline data set and at least 1 reassessment of cognitive function (eFigure 1 in the Supplement), including 9254 from the ELSA (mean [SD] age, 64.6 [9.8] years; 55.9% [5174 of 9254] women; median follow-up duration, 8 [IQR, 6-8] years) and 10 811 from the CHARLS (mean [SD] age, 57.8 [9.0] years; 50.2% [5425 of 10 811] men; median follow-up duration, 4 [IQR, 4-4] years). Baseline sleep duration and the participant covariates are listed in Table 1.

**Table 1.** Characteristics of Participants in the 2 Independent Studies at Baseline

Characteristic	No. (%)			
	ELSA (n = 9254)		CHARLS (n = 10 811)	
	Men (n = 4080)	Women (n = 5174)	Men (n = 5425)	Women (n = 5386)
Age, mean (SD), y	64.8 (9.2)	64.4 (10.2)	58.9 (8.9)	56.6 (9.0)
Sleep duration per night, h				
≤4	142 (3.5)	300 (5.8)	679 (12.5)	868 (16.1)
5	305 (7.5)	489 (9.5)	696 (12.8)	727 (13.5)
6	809 (19.8)	1035 (20.0)	1197 (22.1)	1141 (21.2)
7	1418 (34.8)	1593 (30.8)	1132 (20.9)	1113 (20.7)
8	1152 (28.2)	1402 (27.1)	1291 (23.8)	1125 (20.9)
9	190 (4.7)	262 (5.1)	209 (3.9)	193 (3.6)
≥10	64 (1.6)	93 (1.8)	221 (4.1)	219 (4.1)
Body mass index, mean (SD)^a^	28.3 (4.6)	28.5 (5.7)	23.1 (3.5)	24.2 (3.8)
Blood pressure, mean (SD), mm Hg				
Systolic	135.2 (16.9)	132.2 (18.5)	130.3 (20.3)	129.0 (21.6)
Diastolic	75.9 (11.0)	74.8 (10.8)	76.6 (12.3)	75.4 (11.8)
High level of education	2053 (50.3)	1854 (35.8)	932 (17.2)	569 (10.6)
Center for Epidemiologic Studies Depression Scale score, median (IQR)	0 (0-1)	1 (0-2)	6 (3-10)	8 (4-13)
Depressive symptoms	400 (9.8)	884 (17.1)	1094 (20.2)	1628 (30.2)
Living alone	965 (23.7)	1936 (37.4)	426 (7.9)	634 (11.8)
Current smoking	565 (13.8)	729 (14.1)	4014 (74.0)	413 (7.7)
Alcohol consumption at least once per week	2654 (65.0)	2445 (47.3)	1745 (32.2)	160 (3.0)
Hypertension	2350 (57.6)	2682 (51.8)	2035 (37.5)	2033 (37.7)
Diabetes	396 (9.7)	338 (6.5)	281 (5.2)	334 (6.2)
Coronary heart disease	355 (8.7)	245 (4.7)	524 (9.7)	739 (13.7)
Stroke	104 (2.5)	118 (2.3)	103 (1.9)	86 (1.6)
Cancer	79 (1.9)	100 (1.9)	27 (0.5)	64 (1.2)
Chronic lung disease	119 (2.9)	118 (2.3)	618 (11.4)	431 (8.0)
Asthma	254 (6.2)	446 (8.6)	213 (3.9)	138 (2.6)
Memory score, mean (SD)	10.3 (3.4)	11.0 (3.5)	15.4 (4.6)	15.3 (4.8)
Executive score, mean (SD) or median (IQR)^b^	21.4 (6.8)	21.0 (6.6)	7 (4-8)	5 (3-8)
Orientation score, median (IQR)	4 (4-4)	4 (4-4)	4 (3-4)	3 (2-4)

Abbreviations: CHARLS, China Health and Retirement Longitudinal Study; ELSA, English Longitudinal Study of Ageing; IQR, interquartile range.

^a^ Calculated as weight in kilograms divided by height in meters squared.

^b^ The executive scores are presented as mean (SD) for the ELSA and as median (IQR) for the CHARLS.

### Baseline Sleep Duration and Cognitive Scores Cross-sectional Analyses

**Figure 1.**
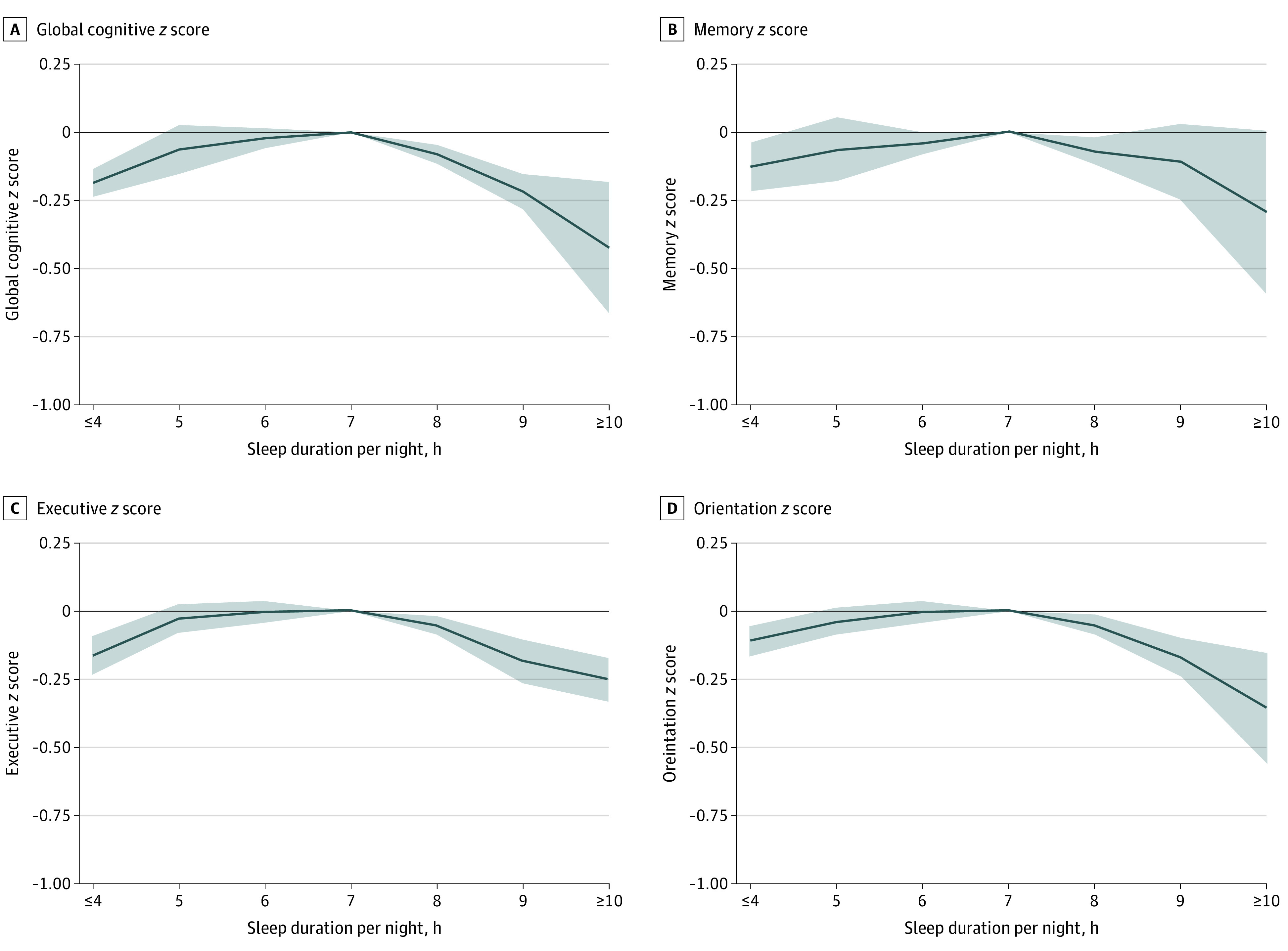
Cross-sectional Associations Between Sleep Duration per Night and *z* Scores at Baseline A-D, Participants who had a sleep duration of 7 hours per night served as the reference group. Solid lines represent adjusted least-squares means after adjusting for sex, age, body mass index, systolic blood pressure, level of education, Center for Epidemiologic Studies Depression Scale score, cohabitation status, current smoking, alcohol consumption, diabetes, coronary heart disease, stroke, cancer, chronic lung disease, and asthma. The shaded areas represent the 95% CIs. Detailed results are listed in Table 2 and eTables 1, 2, and 3 in the Supplement.

As summarized in Table 2, the results derived from the 2 cohorts were comparable. The adjusted least-squares means of the global cognitive *z* scores among individuals reporting 8, 9, 10 or more, or 4 hours or fewer of sleep per night were statistically significantly lower than in the reference group (7 hours per night). Similarly, a statistically significant association between longer (8, 9, or ≥10 hours) or shorter (≤4 hours) sleep duration and lower baseline scores in the 3 cognitive domains was observed except for that between sleep duration of 9 or 10 hours or more and memory function (eTables 1, 2, and 3 in the Supplement). Figure 1 shows the results from the pooled analyses, in which inverted U-shaped associations between baseline sleep duration and the 4 cognitive *z* scores were observed after adjusting for covariates. The individuals who slept 10 hours or more per night had the lowest cognitive scores (Figure 1).

**Table 2.** Cross-Sectional Association Between Sleep Duration per Night and Global Cognitive Function at Baseline Using Analyses of Covariance

Sleep duration per night, h	ELSA (n = 9254)		CHARLS (n = 10 811)		Pooled analysis (N = 20 065)			
	LSM (95% CI) of global *z* scores^a^	*P* value	LSM (95% CI) of global *z* scores^a^	*P* value	Pooled LSM (95% CI) of global *z* scores	*P* value^b^	*I*^2^ statistic, %	*P* value^c^
≤4	−0.19 (−0.28 to −0.09)	<.001	−0.19 (−0.25 to −0.12)	<.001	−0.19 (−0.24 to −0.13)	<.001	0.0	.99
5	−0.11 (−0.18 to −0.04)	.002	−0.02 (−0.08 to 0.04)	.55	−0.06 (−0.15 to 0.03)	.17	73.2	.05
6	−0.03 (−0.09 to 0.02)	.19	−0.01 (−0.06 to 0.05)	.78	−0.02 (−0.06 to 0.02)	.26	0.0	.48
7	1 [Reference]	NA	1 [Reference]	NA	1 [Reference]	NA	NA	NA
8	−0.09 (−0.14 to −0.05)	<.001	−0.07 (−0.12 to −0.01)	.01	−0.08 (−0.12 to −0.05)	<.001	0.0	.46
9	−0.22 (−0.31 to −0.14)	<.001	−0.21 (−0.31 to −0.11)	<.001	−0.22 (−0.28 to −0.15)	<.001	0.0	.84
≥10	−0.55 (−0.69 to −0.41)	<.001	−0.31 (−0.40 to −0.21)	<.001	−0.42 (−0.66 to −0.18)	.001	87.7	.004

Abbreviations: CHARLS, China Health and Retirement Longitudinal Study; ELSA, English Longitudinal Study of Ageing; LSM, least-squares means; NA, not applicable.

^a^ After adjusting for sex, age, body mass index, systolic blood pressure, level of education, Center for Epidemiologic Studies Depression Scale score, cohabitation status, current smoking, alcohol consumption, diabetes, coronary heart disease, stroke, cancer, chronic lung disease, and asthma.

^b^ For the pooled LSM of global *z* scores.

^c^ For the *I*^2^ statistic.

### Baseline Sleep Duration and Cognitive Decline Longitudinal Analyses

**Figure 2.**
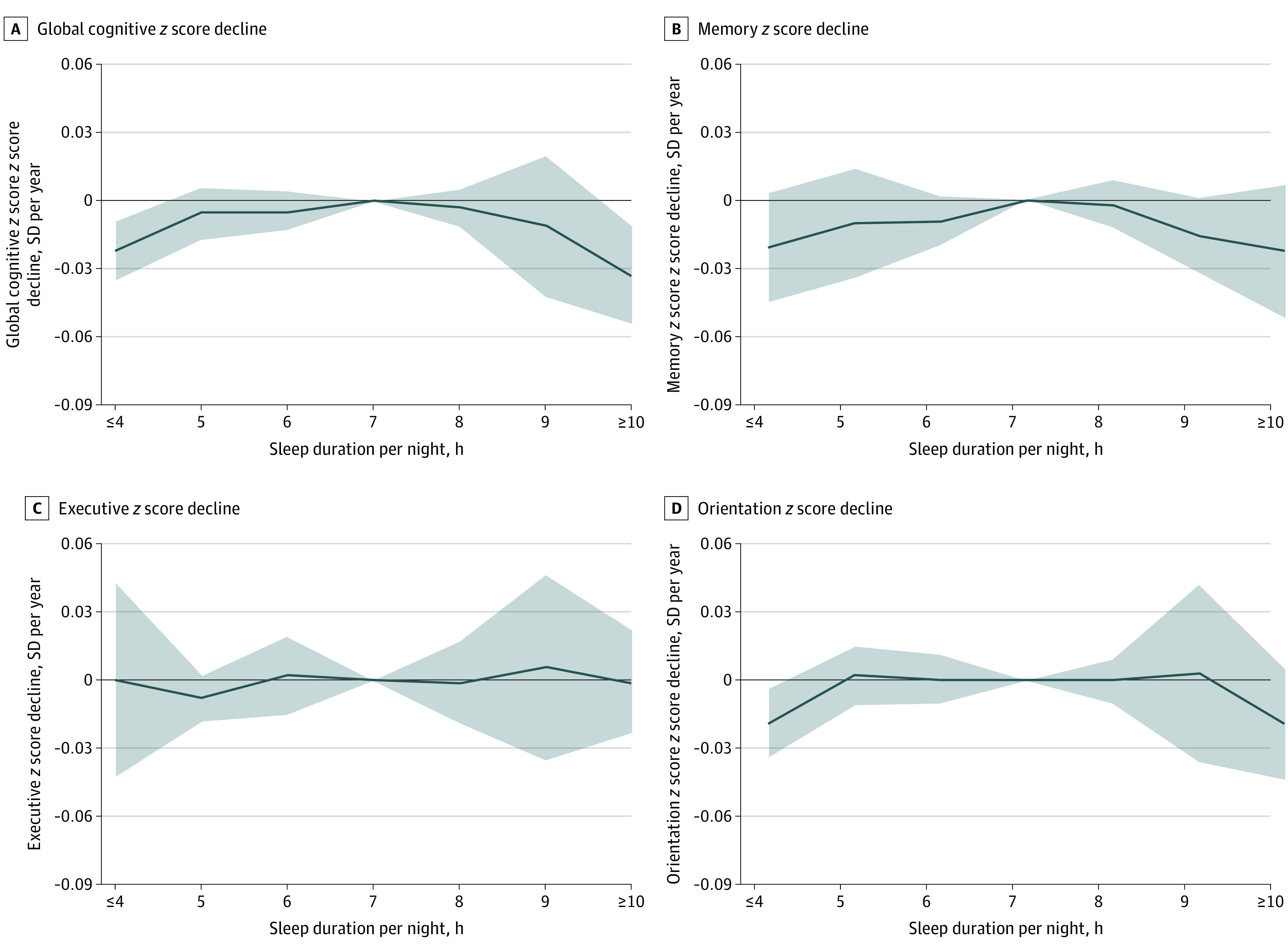
Mean Differences in Rate of Change in *z* Scores During Follow-up A-D, Participants who had a sleep duration of 7 hours per night served as the reference group. Solid lines represent adjusted mean differences after adjusting for sex, age, body mass index, systolic blood pressure, level of education, Center for Epidemiologic Studies Depression Scale score, cohabitation status, current smoking, alcohol consumption, diabetes, coronary heart disease, stroke, cancer, chronic lung disease, and asthma. The shaded areas represent the 95% CIs. Detailed results are listed in Table 3 and eTables 4, 5, and 6 in the Supplement.

Table 3 lists the longitudinal results of the 2 cohorts and the pooled estimates of global cognitive decline. During 100 000 person-years of follow-up, global cognitive *z* scores in individuals with 4 hours or less (pooled β = −0.022; 95% CI, −0.035 to −0.009 SD per year; *P* = .001) and 10 hours or more (pooled β = −0.033; 95% CI, −0.054 to −0.011 SD per year; *P* = .003) of sleep per night declined faster than in the reference group (7 hours per night) after adjusting for a number of covariates. The *I*^2^ statistic indicated that no statistically significant heterogeneity between cohorts was observed (Table 3). An inverted U-shaped association between sleep duration and global cognitive decline, as well as memory, was observed (Figure 2) after adjusting for covariates. A sleep duration of 4 hours or less was statistically significantly associated with faster orientation decline (pooled β = −0.019; 95% CI, −0.034 to −0.004 SD per year; *P* = .01) but not decline in memory (pooled β = −0.021; 95% CI, −0.045 to 0.003 SD per year; *P* = .09) or executive function (pooled β = 0.000; 95% CI, −0.042 to 0.043 SD per year; *P* > .99) (Figure 2 and eTables 4, 5, and 6 in the Supplement). Associations between sleep duration of 10 hours or more and single domains did not achieve statistical significance for memory (pooled β = −0.022; 95% CI, −0.052 to 0.007 SD per year; *P* = .14), executive function (pooled β = −0.001; 95% CI, −0.023 to 0.022 SD per year; *P* = .96), or orientation (pooled β = −0.019; 95% CI, −0.044 to 0.005 SD per year; *P* = .12) (Figure 2 and eTables 4, 5, and 6 in the Supplement).

**Table 3.** Mean Difference in Rate of Change in Global Cognitive Decline (SD per Year) During Follow-up Using Linear Mixed Models

Sleep duration per night, h	ELSA (n = 9254)		CHARLS (n = 10 811)		Pooled analysis (N = 20 065)			
	β (95% Cl)^a^	*P* value	β (95% Cl)^a^	*P* value	Pooled β (95% Cl)	*P* value^b^	*I*^2^ statistic, %	*P* value^c^
≤4	−0.021 (−0.038 to −0.003)	.02	−0.024 (−0.044 to −0.003)	.02	−0.022 (−0.035 to −0.009)	.001	0	.83
5	−0.004 (−0.018 to 0.009)	.51	−0.007 (−0.028 to 0.014)	.51	−0.005 (−0.017 to 0.006)	.37	0	.84
6	−0.005 (−0.015 to 0.005)	.33	−0.003 (−0.022 to 0.015)	.71	−0.005 (−0.013 to 0.004)	.30	0	.89
7	1 [Reference]	NA	1 [Reference]	NA	1 [Reference]	NA	NA	NA
8	−0.004 (−0.012 to 0.005)	.45	−0.001 (−0.020 to 0.017)	.88	−0.003 (−0.011 to 0.005)	.45	0	.85
9	−0.023 (−0.041 to −0.006)	.007	0.009 (−0.025 to 0.042)	.61	−0.011 (−0.042 to 0.020)	.49	64.4	.09
≥10	−0.030 (−0.059 to −0.001)	.05	−0.036 (−0.068 to −0.004)	.03	−0.033 (−0.054 to −0.011)	.003	0	.78

Abbreviations: CHARLS, China Health and Retirement Longitudinal Study; ELSA, English Longitudinal Study of Ageing; NA, not applicable.

^a^ After adjusting for sex, age, body mass index, systolic blood pressure, level of education, Center for Epidemiologic Studies Depression Scale score, cohabitation status, current smoking, alcohol consumption, diabetes, coronary heart disease, stroke, cancer, chronic lung disease, and asthma.

^b^ For the pooled β.

^c^ For the *I*^2^ statistic.

### Nonresponse Analyses

From the baseline population, 1215 of 10 469 individuals (11.6%) from the ELSA and 1264 of 12 075 individuals (10.5%) from the CHARLS were excluded from the analyses because of loss to follow-up. In both cohorts, compared with the included participants, the excluded participants were older, had higher systolic blood pressure, were more likely to live alone, were less likely to engage in alcohol consumption, had higher rates of self-reported coronary heart disease and stroke, and had lower memory and orientation scores at baseline (eTables 7 and 8 in the Supplement).

### Sensitivity Analyses

To assess the potential impact of participant sex on the main results, separate analyses were performed by sex. The results also showed an inverted U-shaped association between sleep duration and global cognitive function and a subsequent decline in both men and women, similar to the main analyses. However, some associations, especially those in the longitudinal analyses, were no longer statistically significant for single domains (eTables 9, 10, 11, 12, 13, 14, 15, 16, 17, 18, 19, 20, 21, 22, 23, and 24 and eFigures 2, 3, 4, and 5 in the Supplement).

## Discussion

In this pooled cohort study of large groups from the ELSA and the CHARLS, a statistically significant inverted U-shaped association was observed between sleep duration and cognitive function. We also found that extreme sleep duration at baseline, including 4 hours or less or 10 hours or more per night, was statistically significantly associated with faster cognitive decline during 100 000 person-years of follow-up. These findings were consistent in both the ELSA and the CHARLS despite the substantial cultural and racial/ethnic differences. To the best of our knowledge, the present study is one of the largest cohort studies to analyze the association between sleep duration and subsequent cognitive decline by combining 2 nationally representative aging cohorts.

Previous studies^7,8,9,10,11,12,13,14,15,16,17,18,19,20,21,22,23,24^ have investigated the longitudinal association between sleep duration and cognitive decline or dementia. Tworoger et al^12^ analyzed data from the US Nurses’ Health Study. However, they found no association between sleep duration and cognitive function, likely because of the short follow-up period (2 years) and limited sample size (n = 1884). Virta et al^7^ analyzed data from the Finnish Twin Cohort in 2013 and noted that individuals with long (>8 hours per day) or short (<7 hours per day) sleep duration at baseline had lower subsequent cognitive test scores. Although similar findings have been reported by other researchers,^8,9,11^ some studies^12,13,14,15,16,17,18,19,20,21,22,23,24^ do not support this conclusion. Most of these previous studies were conducted using sample sizes of less than 1000 individuals,^14,15,19,20,22,24^ samples of only 1 sex,^8,9,12,14,17,18^ or less than 3 years of follow-up.^12,16,21^ Only 2 studies^8,10^ included more than 10 000 individuals, and both revealed inverted U-shaped associations between sleep duration and subsequent cognitive decline, as well as incident dementia, which is in agreement with the present study. However, these 2 studies were weak in representativeness because they used an all-female cohort^8^ and a twin cohort.^10^

In the present longitudinal study, a differential association was detected between sleep duration and distinct cognitive aspects, and memory was the main cognitive domain altered among the 3 domains measured. Memory impairment is the core symptom of dementia and can be considered a factor in the conversion from mild cognitive impairment to dementia.^42^ Previous studies have indicated that acute sleep deprivation impairs memory encoding and consolidation^43,44^ and that short sleep duration is associated with an increase in the risk of developing memory deficits.^45^ Moreover, epidemiological studies^46,47^ have found that long sleep duration is statistically significantly associated with memory deficits in both middle-aged adults and older participants, even after controlling for comorbidities, anxiety, and depression. These data suggest that memory may be altered by changes in sleep habits.

The mechanisms underlying the association between sleep duration and cognitive decline remain unclear, although several plausible biological pathways have been identified. Although the cerebral cortex normally thins with age, a longitudinal study^48^ reported an association between sleep durations of more or less than 7 hours and increased cortical thinning in the frontotemporal areas among cognitively normal older adults. Patel et al^49^ suggested that activity in the interleukin 6 and C-reactive protein inflammatory pathways could be elevated by excessive sleep duration, and they reported a linear association between interleukin 6, C-reactive protein, and sleep duration. Furthermore, inflammatory disorders have been shown to mediate age-related cognitive impairment.^50^

With respect to short sleep duration, long-term fatigue might be an intermediate variable impacting cognitive decline.^51^ Brief periods of sleep deprivation have been associated with an increase in hippocampal synaptic plasticity, contributing to subsequent impaired cognitive function.^52^ The amyloid cascade hypothesis that regards the deposition of amyloid plaques as a crucial event for the etiology of Alzheimer disease might explain the underlying mechanism of sleep deprivation–induced impairment. A randomized clinical trial^53^ in healthy middle-aged men indicated that even 1 night of sleep deprivation could elevate cerebrospinal fluid levels of Aβ42 protein. Tau, a microtubule-associated protein and another accelerator of Alzheimer disease–related neurodegeneration, has also been found to increase by 50% in human cerebrospinal fluid and by 100% in mouse interstitial fluid during chronic sleep deprivation.^54^

### Strengths and Limitations

The foremost strength of the present study is that an association between sleep duration and cognitive function was detected in 2 large cohorts from different cultures. That our findings were consistent across 2 nationally representative community-based cohorts enhances the generalizability of the data, and the large sample size enables more certainty in our statistical analysis. Fortunately, these 2 cohorts shared the exact same measurement methods for sleep duration, memory, and orientation, which facilitated the pooled analyses. Second, the participants in both cohorts were asked about sleep duration without being given durations to choose from, which was not the case in some prior studies.^8,9,12^ This approach, combined with the large population sizes, contributed to the identification of a reliable association between extreme sleep duration and cognitive decline. In addition, no statistically significant heterogeneity between cohorts was observed in the longitudinal analyses for global cognitive function, indicating that the results from the ELSA and the CHARLS were comparable.

This study has several limitations. First, this investigation was an observational study, so no causal relationships could be demonstrated. There is a possibility of reverse causality. Excessive or short sleep duration might be an early manifestation of brain impairment. Furthermore, individuals with memory impairment might not accurately remember their sleep duration. Second, similar to most of the previous studies,^7,8,9,10,11,12,13,15,16,19,20,21,22,23^ sleep duration was obtained according to self-reported information rather than objective measurement, which might have resulted in random misclassification and may have biased the results toward the null. Third, selection bias may have occurred because 1215 individuals (11.6%) from the ELSA and 1264 individuals (10.5%) from the CHARLS were excluded from the study because of loss to follow-up. The results from the nonresponse analyses indicate that our study samples were healthier than the initial samples from the ELSA and the CHARLS. However, loss to follow-up of less than 20% of the overall sample is not considered to imply the presence of considerable bias, even when the missing participants are not missing at random.^55^ Fourth, although we adjusted for a number of confounding factors in our analyses, unmeasured covariates might still have led to confounding bias, including *APOE* status, other sleep disorders, and medication use. Fifth, cognitive function was measured in both cohorts using isolated tasks. These might not have been sufficiently sensitive to detect subtle declines in cognitive function over the follow-up period, thus biasing the results toward the null.

## Conclusions

A statistically significant inverted U-shaped association was observed between sleep duration and cognitive function, as well as subsequent decline. Extreme sleep duration (ie, ≤4 or ≥10 hours per night) was associated not only with lower cognitive function at baseline but also with faster cognitive decline during the follow-up assessments. The inverted U-shaped association indicates that cognitive function should be monitored in middle-aged and older individuals with insufficient or excessive sleep duration. Future mechanism studies and intervention studies examining the association between sleep duration and cognitive decline are needed.

## Notes

Supplement. **eMethods.** Supplemental Methods **eTable 1.** Association Between Sleep Duration per Night and Memory Function at Baseline **eTable 2.** Association Between Sleep Duration per Night and Executive Function at Baseline **eTable 3.** Association Between Sleep Duration per Night and Orientation Function at Baseline **eTable 4.** Association Between Sleep Duration and Memory Decline (SD/year) During Follow-up **eTable 5.** Association Between Sleep Duration and Executive Decline (SD/year) During Follow-up **eTable 6.** Association Between Sleep Duration and Orientation Decline (SD/year) During Follow-up **eTable 7.** Comparison of Baseline Characteristics Between Participants Included (n = 9254) and Excluded From the ELSA, Due to Loss to Follow-up (n = 1215) **eTable 8.** Comparison of Baseline Characteristics Between the Participants Included (n = 10 811) and Excluded From the CHARLS Due to Loss to Follow-up (n = 1264) **eTable 9.** Association Between Sleep Duration per Night and Global Cognitive Function at Baseline in Men **eTable 10.** Association Between Sleep Duration per Night and Memory Function at Baseline in Men **eTable 11.** Association Between Sleep Duration per Night and Executive Function at Baseline in Men **eTable 12.** Association Between Sleep Duration per Night and Orientation Function at Baseline in Men **eTable 13.** Association Between Sleep Duration per Night and Global Cognitive Function at Baseline in Women **eTable 14.** Association Between Sleep Duration per Night and Memory Function at Baseline in Women **eTable 15.** Association Between Sleep Duration per Night and Executive Function at Baseline in Women **eTable 16.** Association Between Sleep Duration per Night and Orientation Function at Baseline in Women **eTable 17.** Association Between Sleep Duration and Global Cognitive Decline (SD/y) During Follow-up in Men **eTable 18.** Association Between Sleep Duration and Memory Decline (SD/year) During Follow-up in Men **eTable 19.** Association Between Sleep Duration and Executive Decline (SD/year) During Follow-up in Men **eTable 20.** Association Between Sleep Duration and Orientation Decline (SD/year) During Follow-up in Men **eTable 21.** Association Between Sleep Duration and Global Cognitive Decline (SD/y) During Follow-up in Women **eTable 22.** Association Between Sleep Duration and Memory Decline (SD/year) During Follow-up in Women **eTable 23.** Association Between Sleep Duration and Executive Decline (SD/year) During Follow-up in Women **eTable 24.** Association Between Sleep Duration and Orientation Decline (SD/year) During Follow-up in Women **eFigure 1.** Flow Chart of Participant Selection for This Study **eFigure 2.** Cross-sectional Associations Between Sleep Duration per Night and Cognitive Functions at Baseline in Men **eFigure 3.** Cross-sectional Associations Between Sleep Duration per Night and Cognitive Functions at Baseline in Women **eFigure 4.** Longitudinal Associations Between Sleep Duration per Night and Cognitive Declines During Follow-up in Men **eFigure 5.** Longitudinal Associations Between Sleep Duration per Night and Cognitive Declines During Follow-up in Women
