# Supplementary material for: Association Between Sleep Duration and Cognitive Decline
Source: JAMA Netw Open. 2020 Sep 21;3(9):e2013573. doi: 10.1001/jamanetworkopen.2020.13573 (PMC7506513; doi:10.1001/jamanetworkopen.2020.13573)
Supplement: Supplement. — eMethods. Supplemental Methods eTable 1. Association Between Sleep Duration per Night and Memory Function at Baseline eTable 2. Association Between Sleep Duration per Night and Executive Function at Baseline eTable 3. Association Between Sleep Duration per Night and Orientation Function at Baseline eTable 4. Association Between Sleep Duration and Memory Decline (SD/year) During Follow-up eTable 5. Association Between Sleep Duration and Executive Decline (SD/year) During Follow-up eTable 6. Association Between Sleep Duration and Orientation Decline (SD/year) During Follow-up eTable 7. Comparison of Baseline Characteristics Between Participants Included (n = 9254) and Excluded From the ELSA, Due to Loss to Follow-up (n = 1215) eTable 8. Comparison of Baseline Characteristics Between the Participants Included (n = 10 811) and Excluded From the CHARLS Due to Loss to Follow-up (n = 1264) eTable 9. Association Between Sleep Duration per Night and Global Cognitive Function at Baseline in Men eTable 10. Association Between Sleep Duration per Night and Memory Function at Baseline in Men eTable 11. Association Between Sleep Duration per Night and Executive Function at Baseline in Men eTable 12. Association Between Sleep Duration per Night and Orientation Function at Baseline in Men eTable 13. Association Between Sleep Duration per Night and Global Cognitive Function at Baseline in Women eTable 14. Association Between Sleep Duration per Night and Memory Function at Baseline in Women eTable 15. Association Between Sleep Duration per Night and Executive Function at Baseline in Women eTable 16. Association Between Sleep Duration per Night and Orientation Function at Baseline in Women eTable 17. Association Between Sleep Duration and Global Cognitive Decline (SD/y) During Follow-up in Men eTable 18. Association Between Sleep Duration and Memory Decline (SD/year) During Follow-up in Men eTable 19. Association Between Sleep Duration and Executive Decline (SD/year) During Follow-up i [file jamanetwopen-e2013573-s001.pdf]

Supplementary Online Content

Ma Y, Liang L, Zheng F, Shi L, Zhong B, Xie W. Association between sleep duration and cognitive decline. *JAMA Netw Open*. 2020;3(9):e2013573.
doi:10.1001/jamanetworkopen.2020.13573

eMethods. Supplemental Methods
eTable 1. Association Between Sleep Duration per Night and Memory Function at Baseline
eTable 2. Association Between Sleep Duration per Night and Executive Function at Baseline
eTable 3. Association Between Sleep Duration per Night and Orientation Function at Baseline
eTable 4. Association Between Sleep Duration and Memory Decline (SD/year) During Follow-up
eTable 5. Association Between Sleep Duration and Executive Decline (SD/year) During Follow-up
eTable 6. Association Between Sleep Duration and Orientation Decline (SD/year) During Follow-up
eTable 7. Comparison of Baseline Characteristics Between Participants Included (n=9254) and Excluded From the ELSA, Due to Loss to Follow-up (n=1215)
eTable 8. Comparison of Baseline Characteristics Between the Participants Included (n=10 811) and Excluded From the CHARLS Due to Loss to Follow-up (n=1264)
eTable 9. Association Between Sleep Duration per Night and Global Cognitive Function at Baseline in Men
eTable 10. Association Between Sleep Duration per Night and Memory Function at Baseline in Men
eTable 11. Association Between Sleep Duration per Night and Executive Function at Baseline in Men
eTable 12. Association Between Sleep Duration per Night and Orientation Function at Baseline in Men
eTable 13. Association Between Sleep Duration per Night and Global Cognitive Function at Baseline in Women
eTable 14. Association Between Sleep Duration per Night and Memory Function at Baseline in Women
eTable 15. Association Between Sleep Duration per Night and Executive Function at Baseline in Women
eTable 16. Association Between Sleep Duration per Night and Orientation Function at Baseline in Women
eTable 17. Association Between Sleep Duration and Global Cognitive Decline (SD/year) During Follow-up in Men
eTable 18. Association Between Sleep Duration and Memory Decline (SD/year) During Follow-up in Men
eTable 19. Association Between Sleep Duration and Executive Decline (SD/year) During Follow-up in Men
eTable 20. Association Between Sleep Duration and Orientation Decline (SD/year) During

© 2020 Ma Y et al. *JAMA Network Open*.

Follow-up in Men
eTable 21. Association Between Sleep Duration and Global Cognitive Decline (SD/year) During Follow-up in Women
eTable 22. Association Between Sleep Duration and Memory Decline (SD/year) During Follow-up in Women
eTable 23. Association Between Sleep Duration and Executive Decline (SD/year) During Follow-up in Women
eTable 24. Association Between Sleep Duration and Orientation Decline (SD/year) During Follow-up in Women
eFigure 1. Flow Chart of Participant Selection for This Study
eFigure 2. Cross-sectional Associations Between Sleep Duration per Night and Cognitive Functions at Baseline in Men
eFigure 3. Cross-sectional Associations Between Sleep Duration per Night and Cognitive Functions at Baseline in Women
eFigure 4. Longitudinal Associations Between Sleep Duration per Night and Cognitive Declines During Follow-up in Men
eFigure 5. Longitudinal Associations Between Sleep Duration per Night and Cognitive Declines During Follow-up in Women

This supplementary material has been provided by the authors to give readers additional information about their work.

© 2020 Ma Y et al. *JAMA Network Open*.

eMethods. Supplemental Methods

1. Sleep duration

In the ELSA, individuals were asked to report “How many hours of sleep do you have on an average week night? (You can tell me to the nearest half hour)”.

In the CHARLS, individuals were asked to report “During the past month, how many hours of actual sleep did you get at night (average hours for one night)? (This may be shorter than the number of hours you spend in bed)”.

2. Cognitive assessment

Cognitive assessment was conducted in face-to-face interviews and covered three domains, memory, executive function and orientation. The ELSA and the CHARLS used the same methods to assess memory and orientation.

2.1 Memory

The interviewers read out a set of 10 words “at a slow steady rate approximately one word every 2 seconds” and then ask the individual to “recall aloud as many of the words as you can, in any order”. Enough time was allowed to recall, approximately up to 2 minutes. After questions and tests on other aspects, the individuals were again asked to recall the words as many as they could.

2.2 Executive function

In the ELSA, individuals were asked to “name as many different animals as you can think of” in one minute.

In the CHARLS, individuals were asked to answer “What does 100 minus 7 equal? And 7 from that? And 7 from that? And 7 from that? And 7 from that?” In addition, individuals were requested to see and draw the following picture.

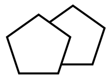

2.3 Orientation

The individuals were asked to report the “today's date (the day of month, month, year)” and “what day of the week it is today”.

3. Covariates

All the covariates were collected in face-to-face interviews unless otherwise stated.

3.1 Age

Individuals were asked to report “date of birth”.

3.2 Sex

Sex were coded with or without asking.

3.3 Education

In the ELSA, individuals reported their “highest educational qualification” in a self-completion

© 2020 Ma Y et al. *JAMA Network Open*.

questionnaire, where education level was classified as no qualification, level 1 national vocational qualification (NVQ) or certificate of secondary education, NVQ2 or general certificate of education (GCE) O-level, NVQ3 or GCE A-level, higher qualification but below degree, and degree level or higher or NVQ4/5. High level of education was defined as ≥ NVQ3 or GCE A-level.

In the CHARLS, individuals reported their "the highest level of education completed" in interview. Education level was classified as no formal education (illiterate), did not finish primary school but capable of reading and/or writing, sishu/home school, elementary school, middle school, high school, vocational school, two-/three-year college/associate degree, four-year college/Bachelor's degree, Master's degree, Doctoral degree/Ph.D. High level of education was defined as ≥ high school.

3.4 Cohabitation status

Cohabitation status indicated currently living alone or not. Individuals in both cohorts were asked to report their "current legal marital status". Common-law marriage was considered as married.

In the ELSA, individuals chose from "single (that is never married), married (first and only marriage), a civil partner in a legally-recognized civil partnership, remarried (second or later marriage), legally separated, divorced, and widowed". Living along was defined as single, legally separated, divorced, or widowed.

In the CHARLS, individuals chose from "married with spouse present, married but not living with spouse temporarily for reasons such as work, separated, divorced, widowed, and never married". Living along was defined as separated, divorced, widowed, or never married.

3.5 The Center for Epidemiologic Studies Depression Scale (CES-D)

The ELSA used an eight-item version of the CES-D. The individuals answered the following eight questions with Yes or No for much of the time during the past week.

(1) You felt depressed?
(2) You felt that everything you did was an effort?
(3) Your sleep was restless?
(4) You were happy?
(5) You felt lonely?
(6) You enjoyed life?
(7) You felt sad?
(8) You could not get going?

The CHARLS instead used a ten-item version of the CES-D as below.

(1) I was bothered by things that don't usually bother me.
(2) I had trouble keeping my mind on what I was doing.
(3) I felt depressed.
(4) I felt everything I did was an effort.
(5) I felt hopeful about the future.
(6) I felt fearful.

© 2020 Ma Y et al. *JAMA Network Open*.

(7) My sleep was restless.
(8) I was happy.
(9) I felt lonely.
(10)I could not get "going."

The individuals were asked to choose one of the following responses for each item above that happened during the last week.

(1) Rarely or none of the time (< 1 day)
(2) Some or a little of the time (1-2 days)
(3) Occasionally or a moderate amount of the time (3-4 days)
(4) Most or all of the time (5-7 days)

3.6 Body mass index

Body mass index was calculated with interviewer-performed physical examination and the following formula: weight (kg) / height^2 (m^2).

3.7 Systolic blood pressure

Systolic blood pressure was the mean of three measures of systolic blood pressure.

3.8 Current smoking

Individuals were asked to answer “Do you smoke cigarettes at all nowadays” in the ELSA or “Do you still chew tobacco, smoke a pipe, smoke self-rolled cigarettes, or smoke cigarettes/cigars?” in the CHARLS with Yes or No.

3.9 Alcohol consumption

Individuals in the ELSA chose the frequency of alcoholic drinking in the past 12 months in a self-completion questionnaire. Twice a day or more, daily or almost daily, once or twice a week, once or twice a month, special occasions only, or, not at all?

In the CHARLS, alcohol consuming involved the following three questions:

(1) How often did you drink liquor, including white liquor, whisky, and others per month in the last year?
(2) How many times per month did you drink beer in the last year?
(3) How often did you drink wine or rice wine per month in the last year?

The individuals were asked to choose one of the following responses for each item above.

(1) Once a month
(2) 2-3 times a month
(3) Once a week
(4) 2-3 times a week
(5) 4-6 times a week
(6) Once a day
(7) Twice a day
(8) More than twice a day

Alcohol consuming in the CHARLS was defined as at least once a week for whichever of the above three questions.

© 2020 Ma Y et al. *JAMA Network Open*.

3.10 Diabetes, coronary heart disease, stroke, cancer, asthma, and chronic lung disease

Individuals were requested to confirm whether they had been told by a doctor that they had the following conditions:

(1) diabetes (diabetes or high blood sugar)

(2) coronary heart disease (angina or heart attack in the ELSA; heart attack, coronary heart disease, angina, congestive heart failure, or other heart problems in the CHARLS)

(3) stroke

(4) cancer (cancer or malignant tumour)

(5) asthma

(6) chronic lung disease (such as chronic bronchitis or emphysema)

In addition, confirmation of current anti-diabetic therapy was included in the definition of diabetes.

© 2020 Ma Y et al. *JAMA Network Open*.

eTable 1. Association between sleep duration per night and memory function at baseline

Sleep duration per night, hours	ELSA (n=9254)			CHARLS（n=10 811)			Pooled analysis (n=20 065)			
	LSM of memory Z scores[a]	*P* value		LSM of memory Z scores[a]	*P* value		Pooled LSM of memory Z scores	*P* value	I^2 (%)	*P* value
≤4	-0.18 (-0.27 to -0.09)	<0.001		-0.09 (-0.16 to -0.02)	.009		-0.13 (-0.22 to -0.04)	0.005	61.1	.11
5	-0.13 (-0.20 to -0.06)	<0.001		-0.01 (-0.07 to 0.06)	.88		-0.06 (-0.18 to 0.05)	0.28	83.4	.01
6	-0.06 (-0.11 to -0.01)	.02		-0.02 (-0.08 to 0.04)	.51		-0.04 (-0.08 to 0.00)	0.05	15.0	.28
7	Reference	/		Reference	/		Reference	/	/	/
8	-0.09 (-0.14 to -0.05)	<0.001		-0.04 (-0.10 to 0.02)	.18		-0.07 (-0.12 to -0.02)	0.01	51.9	.15
9	-0.18 (-0.26 to -0.09)	<0.001		-0.04 (-0.14 to 0.07)	.51		-0.11 (-0.25 to 0.03)	0.13	76.6	.04
≥10	-0.45 (-0.59 to -0.31)	<0.001		-0.14 (-0.24 to -0.04)	.005		-0.29 (-0.59 to 0.01)	0.06	91.6	.001

Abbreviations: ELSA, the English Longitudinal Study of Ageing; CHARLS, the China Health Retirement Longitudinal Study; LSM, least-squares means.

[a] Data are presented as LSM and 95% confidence interval after adjusting for age, sex, education, body mass index, the Center for Epidemiologic Studies Depression Scale score, systolic blood pressure, cohabitation status, current smoking, alcohol consumption, diabetes, coronary heart disease, stroke, cancer, chronic lung disease, and asthma.

© 2020 Ma Y et al. *JAMA Network Open*.

eTable 2. Association between sleep duration per night and executive function at baseline

Sleep duration per night, hours	ELSA (n=9254)		CHARLS（n=10 811)		Pooled analysis (n=20 065)			
	LSM of executive Z scores[a]	*P* value	LSM of executive Z scores[a]	*P* value	Pooled LSM of executive Z scores	*P* value	I^2 (%)	*P* value
≤4	-0.12 (-0.21 to -0.02)	.02	-0.19 (-0.25 to -0.13)	<0.001	-0.16 (-0.24 to -0.09)	<0.001	41.8	.19
5	-0.06 (-0.13 to 0.02)	.12	-0.01 (-0.07 to 0.06)	.85	-0.03 (-0.08 to 0.02)	0.28	11.6	.29
6	-0.01 (-0.07 to 0.04)	.60	0.01 (-0.05 to 0.06)	.82	0.00 (-0.04 to 0.03)	0.84	0.0	.60
7	Reference	/	Reference	/	Reference	/	/	/
8	-0.04 (-0.09 to 0.01)	.11	-0.07 (-0.12 to -0.02)	.01	-0.05 (-0.09 to -0.02)	0.004	0.0	.43
9	-0.15 (-0.24 to -0.05)	.002	-0.23 (-0.32 to -0.13)	<0.001	-0.18 (-0.26 to -0.10)	<0.001	30.1	.23
≥10	-0.27 (-0.42 to -0.12)	<0.001	-0.25 (-0.34 to -0.15)	<0.001	-0.25 (-0.33 to -0.17)	<0.001	0.0	.81

Abbreviations: ELSA, the English Longitudinal Study of Ageing; CHARLS, the China Health Retirement Longitudinal Study; LSM, least-squares means.

[a] Data are presented as LSM and 95% confidence interval after adjusting for age, sex, education, body mass index, the Center for Epidemiologic Studies Depression Scale score, systolic blood pressure, cohabitation status, current smoking, alcohol consumption, diabetes, coronary heart disease, stroke, cancer, chronic lung disease, and asthma.

© 2020 Ma Y et al. *JAMA Network Open*.

eTable 3. Association between sleep duration per night and orientation function at baseline

Sleep duration per night, hours	ELSA (n=9254)		CHARLS (n=10 811)		Pooled analysis (n=20 065)			
	LSM of orientation Z scores[a]	*P* value	LSM of orientation Z scores[a]	*P* value	Pooled LSM of orientation Z scores	*P* value	I^2 (%)	*P* value
≤4	-0.10 (-0.20 to 0.00)	.05	-0.11 (-0.18 to -0.05)	<0.001	-0.11 (-0.17 to -0.06)	<0.001	0.0	.85
5	-0.05 (-0.13 to 0.03)	.18	-0.03 (-0.09 to 0.04)	.38	-0.04 (-0.09 to 0.01)	0.13	0.0	.62
6	0.00 (-0.06 to 0.06)	.95	0.00 (-0.06 to 0.05)	.91	0.00 (-0.04 to 0.04)	0.97	0.0	.90
7	Reference	/	Reference	/	Reference	/	/	/
8	-0.07 (-0.12 to -0.01)	.01	-0.03 (-0.09 to 0.02)	.24	-0.05 (-0.09 to -0.01)	0.009	0.0	.38
9	-0.16 (-0.26 to -0.06)	.001	-0.19 (-0.29 to -0.09)	<0.001	-0.17 (-0.24 to -0.10)	<0.001	0.0	.72
≥10	-0.47 (-0.63 to -0.31)	<0.001	-0.26 (-0.36 to -0.17)	<0.001	-0.36 (-0.56 to -0.15)	0.001	79.7	.03

Abbreviations: ELSA, the English Longitudinal Study of Ageing; CHARLS, the China Health Retirement Longitudinal Study; LSM, least-squares means.

[a] Data are presented as LSM and 95% confidence interval after adjusting for age, sex, education, body mass index, the Center for Epidemiologic Studies Depression Scale score, systolic blood pressure, cohabitation status, current smoking, alcohol consumption, diabetes, coronary heart disease, stroke, cancer, chronic lung disease, and asthma.

© 2020 Ma Y et al. *JAMA Network Open*.

eTable 4. Association between sleep duration and memory decline (SD/year) during follow-up

Sleep duration per night, hours	ELSA (n=9254)			CHARLS（n=10 811)			Pooled analysis (n=20 065)			
	**β (95% CI)**	***P* value[a]**		**β (95% CI)**	***P* value[a]**		**Pooled β (95% CI)**	***P* value**	**I^2 (%)**	***P* value**
≤4	-0.011 (-0.024 to 0.003)	.12		-0.035 (-0.059 to -0.012)	.003		-0.021 (-0.045 to 0.003)	0.09	69.7	.07
5	0.000 (-0.010 to 0.010)	.99		-0.025 (-0.049 to -0.001)	.04		-0.010 (-0.034 to 0.014)	0.41	71.9	.06
6	-0.006 (-0.013 to 0.002)	.15		-0.019 (-0.040 to 0.002)	.08		-0.009 (-0.019 to 0.002)	0.13	28.7	.24
7	Reference	/		Reference	/		Reference	/	/	/
8	0.001 (-0.005 to 0.008)	.68		-0.012 (-0.033 to 0.009)	.26		-0.002 (-0.012 to 0.009)	0.78	31.1	.23
9	-0.012 (-0.025 to 0.001)	.07		-0.034 (-0.072 to 0.004)	.08		-0.016 (-0.032 to 0.001)	0.06	16.3	.27
≥10	-0.011 (-0.033 to 0.011)	.35		-0.041 (-0.078 to -0.005)	.03		-0.022 (-0.052 to 0.007)	0.14	50.1	.16

Abbreviations: ELSA, the English Longitudinal Study of Ageing; CHARLS, the China Health Retirement Longitudinal Study.

[a] After adjusting for age, sex, education, body mass index, the Center for Epidemiologic Studies Depression Scale score, systolic blood pressure, cohabitation status, current smoking, alcohol consumption, diabetes, coronary heart disease, stroke, cancer, chronic lung disease, and asthma.

© 2020 Ma Y et al. *JAMA Network Open*.

eTable 5. Association between sleep duration and executive decline (SD/year) during follow-up

Sleep duration per night, hours	ELSA (n=9254)			CHARLS (n=10 811)			Pooled analysis (n=20 065)			
	β (95% CI)	*P* value[a]		β (95% CI)	*P* value[a]		Pooled β (95% CI)	*P* value	I^2 (%)	*P* value
≤4	-0.021 (-0.035 to -0.007)	.003		0.022 (0.006 to 0.038)	.009		0.000 (-0.042 to 0.043)	1.00	93.6	<0.001
5	-0.011 (-0.022 to 0.000)	.04		0.000 (-0.017 to 0.017)	.99		-0.008 (-0.018 to 0.002)	0.14	15.4	.28
6	-0.005 (-0.013 to 0.003)	.18		0.012 (-0.003 to 0.026)	.11		0.002 (-0.015 to 0.019)	0.80	76.3	.04
7	Reference	/		Reference	/		Reference	/	/	/
8	-0.009 (-0.016 to -0.002)	.01		0.010 (-0.005 to 0.024)	.19		-0.001 (-0.019 to 0.017)	0.94	80.6	.02
9	-0.013 (-0.027 to 0.000)	.05		0.028 (0.002 to 0.055)	.04		0.006 (-0.035 to 0.046)	0.78	86.7	.006
≥10	-0.011 (-0.035 to 0.012)	.33		0.012 (-0.014 to 0.038)	.38		-0.001 (-0.023 to 0.022)	0.96	41.2	.19

Abbreviations: ELSA, the English Longitudinal Study of Ageing; CHARLS, the China Health Retirement Longitudinal Study.

[a] After adjusting for age, sex, education, body mass index, the Center for Epidemiologic Studies Depression Scale score, systolic blood pressure, cohabitation status, current smoking, alcohol consumption, diabetes, coronary heart disease, stroke, cancer, chronic lung disease, and asthma.

© 2020 Ma Y et al. *JAMA Network Open*.

eTable 6. Association between sleep duration and orientation decline (SD/year) during follow-up

Sleep duration per night, hours	ELSA (n=9254)			CHARLS（n=10 811)			Pooled analysis (n=20 065)			
	β (95% CI)	*P* value[a]		β (95% CI)	*P* value[a]		Pooled β (95% CI)	*P* value	I^2 (%)	*P* value
≤4	-0.019 (-0.041 to 0.003)	.10		-0.020 (-0.041 to 0.001)	.06		-0.019 (-0.034 to -0.004)	0.01	0.0	.94
5	-0.001 (-0.018 to 0.016)	.90		0.007 (-0.014 to 0.028)	.52		0.002 (-0.011 to 0.015)	0.76	0.0	.56
6	-0.002 (-0.014 to 0.010)	.75		0.005 (-0.014 to 0.024)	.60		0.000 (-0.010 to 0.011)	0.98	0.0	.54
7	Reference	/		Reference	/		Reference	/	/	/
8	0.000 (-0.012 to 0.011)	.95		-0.001 (-0.019 to 0.018)	.93		0.000 (-0.010 to 0.009)	0.92	0.0	.97
9	-0.015 (-0.037 to 0.006)	.17		0.025 (-0.009 to 0.059)	.15		0.003 (-0.036 to 0.042)	0.89	73.9	.05
≥10	-0.022 (-0.059 to 0.014)	.23		-0.017 (-0.049 to 0.016)	.31		-0.019 (-0.044 to 0.005)	0.12	0.0	.82

Abbreviations: ELSA, the English Longitudinal Study of Ageing; CHARLS, the China Health Retirement Longitudinal Study.

[a] After adjusting for age, sex, education, body mass index, the Center for Epidemiologic Studies Depression Scale score, systolic blood pressure, cohabitation status, current smoking, alcohol consumption, diabetes, coronary heart disease, stroke, cancer, chronic lung disease, and asthma.

© 2020 Ma Y et al. *JAMA Network Open*.

eTable 7. Comparison of baseline characteristics between participants included (n=9254) and excluded from the ELSA, due to loss to follow-up (n=1215)

Characteristic	Included (n=9254)	Loss to follow-up (n=1215)	*P* for difference[a]
Age (years)	64.6±9.8	69.0±12.9	<0.001
Male (%)	4080 (44.1)	553 (45.5)	0.35
Sleep duration per night, hours (%)			
≤4	442 (4.8)	75 (6.2)	<0.001
5	794 (8.6)	116 (9.6)	
6	1844 (19.9)	243 (20.0)	
7	3011 (32.5)	327 (26.9)	
8	2554 (27.6)	318 (26.2)	
9	452 (4.9)	75 (6.2)	
≥10	157 (1.7)	61 (5.0)	
Body mass index (kg/m^2)	28.4±5.3	27.9±5.6	0.004
Systolic blood pressure (mm Hg)	133.3±18.0	134.1±19.8	0.18
Diastolic blood pressure (mm Hg)	75.2±10.9	73.4±12.2	<0.001
High level of education (%)	3907 (42.2)	381 (31.4)	<0.001
The Center for Epidemiologic Studies Depression Scale scores	1 (0–2)	1 (0–3)	<0.001
Depressive symptoms (%)	1284 (13.9)	233 (19.2)	<0.001
Living alone (%)	2901 (31.4)	470 (38.7)	<0.001
Current smoking (%)	1294 (14.0)	208 (17.1)	0.003
Alcoholic drink ≥once per week (%)	5099 (55.1)	470 (38.7)	<0.001
Hypertension (%)	5019 (54.2)	702 (57.8)	0.02
Diabetes (%)	734 (7.9)	116 (9.6)	0.05
Coronary heart disease (%)	600 (6.5)	134 (11.0)	<0.001
Stroke (%)	222 (2.4)	61 (5.0)	<0.001
Cancer (%)	179 (1.9)	32 (2.6)	0.10
Chronic lung disease (%)	237 (2.6)	41 (3.4)	0.10
Asthma (%)	700 (7.6)	94 (7.7)	0.83

© 2020 Ma Y et al. *JAMA Network Open*.

Memory scores	10.7±3.5	8.6±4.1	<0.001
Executive scores	21.2±6.7	17.8±7.3	<0.001
Orientation scores	4 (4–4)	4 (3–4)	<0.001

Data are presented as mean ± SD, n (%), or median (quartile 1–quartile 3).

[a] Calculated by using a t test, chi-square test, or Wilcoxon rank test.

© 2020 Ma Y et al. *JAMA Network Open*.

eTable 8. Comparison of baseline characteristics between the participants included (n=10 811) and excluded from the CHARLS due to loss to follow-up (n=1264)

Characteristic	Included (n=10811)	Loss to follow-up (n=1264)	*P* for difference[a]
Age (years)	57.8±9.0	60.5±11.4	<0.001
Male (%)	5425 (50.2)	664 (52.5)	0.11
Sleep duration per night, hours (%)			
≤4	1547 (14.3)	159 (12.6)	0.11
5	1423 (13.2)	157 (12.4)	
6	2338 (21.6)	304 (24.1)	
7	2245 (20.8)	252 (19.9)	
8	2416 (22.4)	274 (21.7)	
9	402 (3.7)	53 (4.2)	
≥10	440 (4.1)	65 (5.1)	
Body mass index (kg/m^2)	23.7±3.7	23.5±3.8	0.09
Systolic blood pressure (mm Hg)	129.7±21.1	132.7±22.3	<0.001
Diastolic blood pressure (mm Hg)	76.0±12.1	77.4±12.4	<0.001
High level of education (%)	1501 (13.9)	281 (22.2)	<0.001
The Center for Epidemiologic Studies Depression Scale scores	7 (3–12)	6 (3–12)	0.42
Depressive symptoms (%)	2722 (25.2)	319 (25.2)	0.96
Living alone (%)	1069 (9.9)	188 (14.9)	<0.001
Current smoking (%)	4427 (41.0)	545 (43.1)	0.14
Alcoholic drink ≥once per week (%)	1905 (17.6)	182 (14.4)	0.004
Hypertension (%)	4068 (37.6)	522 (41.3)	0.01
Diabetes (%)	615 (5.7)	83 (6.6)	0.21
Coronary heart disease (%)	1263 (11.7)	176 (13.9)	0.02
Stroke (%)	189 (1.8)	38 (3.0)	0.002
Cancer (%)	91 (0.8)	19 (1.5)	0.02
Chronic lung disease (%)	1049 (9.7)	158 (12.5)	0.002
Asthma (%)	351 (3.3)	56 (4.4)	0.03

© 2020 Ma Y et al. *JAMA Network Open*.

Memory scores	15.4±4.7	14.7±5.4	<0.001
Executive scores	5 (4–8)	5 (4–8)	0.84
Orientation scores	3 (3–4)	4 (3–4)	0.001

Data are presented as mean ± SD, n (%), or median (quartile 1–quartile 3).

[a] Calculated by using a t test, chi-square test, or Wilcoxon rank test.

© 2020 Ma Y et al. *JAMA Network Open*.

eTable 9. Association between sleep duration per night and global cognitive function at baseline in men

Sleep duration per night, hours	ELSA (n=4080)			CHARLS（n=5425)			Pooled analysis (n=9505)			
	LSM of global Z scores[a]	*P* value		LSM of global Z scores[a]	*P* value		Pooled LSM of global Z scores	*P* value	I^2 (%)	*P* value
≤4	-0.19 (-0.35 to -0.03)	.02		-0.19 (-0.27 to -0.10)	<0.001		-0.19 (-0.26 to -0.11)	<0.001	0.0	.99
5	-0.09 (-0.21 to 0.02)	.10		0.04 (-0.04 to 0.12)	.35		-0.02 (-0.15 to 0.11)	0.74	71.4	.06
6	-0.03 (-0.11 to 0.05)	.45		0.03 (-0.04 to 0.10)	.36		0.00 (-0.06 to 0.06)	0.91	26.8	.24
7	Reference	/		Reference	/		Reference	/	/	/
8	-0.07 (-0.14 to 0.00)	.06		-0.05 (-0.12 to 0.02)	.17		-0.06 (-0.11 to -0.01)	0.02	0.0	.69
9	-0.23 (-0.37 to -0.10)	<0.001		-0.10 (-0.23 to 0.02)	.10		-0.17 (-0.29 to -0.04)	0.009	45.6	.18
≥10	-0.25 (-0.48 to -0.03)	.03		-0.27 (-0.39 to -0.15)	<0.001		-0.26 (-0.37 to -0.16)	<0.001	0.0	.90

Abbreviations: ELSA, the English Longitudinal Study of Ageing; CHARLS, the China Health Retirement Longitudinal Study; LSM, least-squares means.

[a] Data are presented as LSM and 95% confidence interval after adjusting for age, education, body mass index, the Center for Epidemiologic Studies Depression Scale score, systolic blood pressure, cohabitation status, current smoking, alcohol consumption, diabetes, coronary heart disease, stroke, cancer, chronic lung disease, and asthma.

© 2020 Ma Y et al. *JAMA Network Open.*

eTable 10. Association between sleep duration per night and memory function at baseline in men

Sleep duration per night, hours	ELSA (n=4080)		CHARLS（n=5425)		Pooled analysis (n=9505)			
	LSM of memory Z scores[a]	*P* value	LSM of memory Z scores[a]	*P* value	Pooled LSM of memory Z scores	*P* value	I^2 (%)	*P* value
≤4	-0.24 (-0.39 to -0.08)	.003	-0.04 (-0.13 to 0.06)	.48	-0.13 (-0.32 to 0.07)	0.21	78.5	.03
5	-0.12 (-0.22 to -0.01)	.04	0.09 (0.00 to 0.18)	.06	-0.01 (-0.21 to 0.19)	0.91	87.2	.005
6	-0.09 (-0.17 to -0.02)	.01	0.04 (-0.04 to 0.12)	.30	-0.03 (-0.16 to 0.11)	0.70	83.2	.02
7	Reference	/	Reference	/	Reference	/	/	/
8	-0.06 (-0.13 to 0.01)	.08	-0.03 (-0.10 to 0.05)	.49	-0.05 (-0.10 to 0.01)	0.08	0.0	.52
9	-0.15 (-0.28 to -0.02)	.03	0.07 (-0.07 to 0.22)	.31	-0.04 (-0.25 to 0.18)	0.73	79.8	.03
≥10	-0.29 (-0.51 to -0.07)	.009	-0.19 (-0.33 to -0.05)	.009	-0.22 (-0.33 to -0.10)	<0.001	0.0	.42

Abbreviations: ELSA, the English Longitudinal Study of Ageing; CHARLS, the China Health Retirement Longitudinal Study; LSM, least-squares means.

[a] Data are presented as LSM and 95% confidence interval after adjusting for age, education, body mass index, the Center for Epidemiologic Studies Depression Scale score, systolic blood pressure, cohabitation status, current smoking, alcohol consumption, diabetes, coronary heart disease, stroke, cancer, chronic lung disease, and asthma.

© 2020 Ma Y et al. *JAMA Network Open*.

eTable 11. Association between sleep duration per night and executive function at baseline in men

Sleep duration per night, hours	ELSA (n=4080)			CHARLS（n=5425)			Pooled analysis (n=9505)			
	LSM of executive Z scores[a]	*P* value		LSM of executive Z scores[a]	*P* value		Pooled LSM of executive Z scores	*P* value	I^2 (%)	*P* value
≤4	0.01 (-0.16 to 0.18)	.94		-0.22 (-0.30 to -0.14)	<0.001		-0.12 (-0.34 to 0.10)	0.29	81.8	.02
5	0.00 (-0.12 to 0.12)	1.00		0.04 (-0.04 to 0.11)	.39		0.02 (-0.04 to 0.09)	0.47	0.0	.64
6	0.02 (-0.06 to 0.10)	.64		0.02 (-0.05 to 0.09)	.54		0.02 (-0.03 to 0.07)	0.44	0.0	.98
7	Reference	/		Reference	/		Reference	/	/	/
8	0.02 (-0.06 to 0.09)	.68		-0.05 (-0.11 to 0.02)	.15		-0.02 (-0.08 to 0.04)	0.56	37.2	.21
9	-0.13 (-0.27 to 0.02)	.08		-0.16 (-0.28 to -0.04)	.01		-0.15 (-0.24 to -0.05)	0.002	0.0	.76
≥10	-0.08 (-0.32 to 0.16)	.52		-0.15 (-0.27 to -0.03)	.01		-0.14 (-0.24 to -0.03)	0.01	0.0	.61

Abbreviations: ELSA, the English Longitudinal Study of Ageing; CHARLS, the China Health Retirement Longitudinal Study; LSM, least-squares means.

[a] Data are presented as LSM and 95% confidence interval after adjusting for age, education, body mass index, the Center for Epidemiologic Studies Depression Scale score, systolic blood pressure, cohabitation status, current smoking, alcohol consumption, diabetes, coronary heart disease, stroke, cancer, chronic lung disease, and asthma.

© 2020 Ma Y et al. *JAMA Network Open*.

eTable 12. Association between sleep duration per night and orientation function at baseline in men

Sleep duration per night, hours	ELSA (n=4080)			CHARLS（n=5425)			Pooled analysis (n=9505)			
	LSM of orientation Z scores[a]	*P* value		LSM of orientation Z scores[a]	*P* value		Pooled LSM of orientation Z scores	*P* value	I^2 (%)	*P* value
≤4	-0.18 (-0.36 to 0.00)	.06		-0.14 (-0.23 to -0.05)	.002		-0.15 (-0.23 to -0.07)	<0.001	0.0	.74
5	-0.09 (-0.21 to 0.04)	.19		-0.04 (-0.13 to 0.04)	.34		-0.06 (-0.13 to 0.02)	0.13	0.0	.57
6	0.01 (-0.08 to 0.10)	.83		0.01 (-0.07 to 0.08)	.89		0.01 (-0.05 to 0.06)	0.81	0.0	.93
7	Reference	/		Reference	/		Reference	/	/	/
8	-0.10 (-0.18 to -0.02)	.01		-0.03 (-0.10 to 0.05)	.48		-0.06 (-0.13 to 0.01)	0.10	45.7	.18
9	-0.22 (-0.38 to -0.07)	.005		-0.14 (-0.27 to 0.00)	.04		-0.17 (-0.27 to -0.07)	0.001	0.0	.40
≥10	-0.17 (-0.42 to 0.09)	.20		-0.23 (-0.36 to -0.11)	<0.001		-0.22 (-0.34 to -0.11)	<0.001	0.0	.65

Abbreviations: ELSA, the English Longitudinal Study of Ageing; CHARLS, the China Health Retirement Longitudinal Study; LSM, least-squares means.

[a] Data are presented as LSM and 95% confidence interval after adjusting for age, education, body mass index, the Center for Epidemiologic Studies Depression Scale score, systolic blood pressure, cohabitation status, current smoking, alcohol consumption, diabetes, coronary heart disease, stroke, cancer, chronic lung disease, and asthma.

© 2020 Ma Y et al. *JAMA Network Open*.

eTable 13. Association between sleep duration per night and global cognitive function at baseline in women

Sleep duration per night, hours	ELSA (n=5174)		CHARLS (n=5386)		Pooled analysis (n=10 560)			
	LSM of global Z scores[a]	*P* value	LSM of global Z scores[a]	*P* value	Pooled LSM of global Z scores	*P* value	I^2 (%)	*P* value
≤4	-0.19 (-0.30 to -0.08)	<0.001	-0.19 (-0.28 to -0.10)	<0.001	-0.19 (-0.26 to -0.12)	<0.001	0.0	.95
5	-0.12 (-0.21 to -0.03)	.008	-0.07 (-0.16 to 0.02)	.12	-0.10 (-0.16 to -0.03)	0.003	0.0	.45
6	-0.04 (-0.11 to 0.03)	.25	-0.04 (-0.12 to 0.04)	.29	-0.04 (-0.09 to 0.01)	0.12	0.0	.96
7	Reference	/	Reference	/	Reference	/	/	/
8	-0.11 (-0.18 to -0.05)	<0.001	-0.08 (-0.16 to 0.00)	.05	-0.10 (-0.15 to -0.05)	<0.001	0.0	.50
9	-0.22 (-0.33 to -0.10)	<0.001	-0.31 (-0.46 to -0.17)	<0.001	-0.25 (-0.35 to -0.16)	<0.001	8.5	.30
≥10	-0.75 (-0.94 to -0.57)	<0.001	-0.34 (-0.48 to -0.20)	<0.001	-0.54 (-0.95 to -0.14)	0.008	91.8	<0.001

Abbreviations: ELSA, the English Longitudinal Study of Ageing; CHARLS, the China Health Retirement Longitudinal Study; LSM, least-squares means.

[a] Data are presented as LSM and 95% confidence interval after adjusting for age, education, body mass index, the Center for Epidemiologic Studies Depression Scale score, systolic blood pressure, cohabitation status, current smoking, alcohol consumption, diabetes, coronary heart disease, stroke, cancer, chronic lung disease, and asthma.

© 2020 Ma Y et al. *JAMA Network Open*.

eTable 14. Association between sleep duration per night and memory function at baseline in women

Sleep duration per night, hours	ELSA (n=5174)			CHARLS（n=5386)			Pooled analysis (n=10 560)			
	LSM of memory Z scores[a]	*P* value		LSM of memory Z scores[a]	*P* value		Pooled LSM of memory Z scores	*P* value	I^2 (%)	*P* value
≤4	-0.16 (-0.27 to -0.05)	.006		-0.14 (-0.24 to -0.05)	.003		-0.15 (-0.22 to -0.08)	<0.001	0.0	.83
5	-0.13 (-0.22 to -0.04)	.005		-0.10 (-0.20 to -0.01)	.04		-0.12 (-0.18 to -0.05)	0.001	0.0	.66
6	-0.04 (-0.11 to 0.03)	.29		-0.08 (-0.17 to 0.00)	.05		-0.06 (-0.11 to 0.00)	0.04	0.0	.41
7	Reference	/		Reference	/		Reference	/	/	/
8	-0.12 (-0.18 to -0.05)	<0.001		-0.05 (-0.13 to 0.04)	.27		-0.09 (-0.16 to -0.02)	0.01	42.8	.19
9	-0.19 (-0.31 to -0.08)	.001		-0.15 (-0.30 to 0.01)	.06		-0.18 (-0.27 to -0.08)	<0.001	0.0	.64
≥10	-0.55 (-0.74 to -0.37)	<0.001		-0.09 (-0.24 to 0.05)	.21		-0.32 (-0.77 to 0.13)	0.17	93.2	<0.001

Abbreviations: ELSA, the English Longitudinal Study of Ageing; CHARLS, the China Health Retirement Longitudinal Study; LSM, least-squares means.

[a] Data are presented as LSM and 95% confidence interval after adjusting for age, education, body mass index, the Center for Epidemiologic Studies Depression Scale score, systolic blood pressure, cohabitation status, current smoking, alcohol consumption, diabetes, coronary heart disease, stroke, cancer, chronic lung disease, and asthma.

© 2020 Ma Y et al. *JAMA Network Open*.

eTable 15. Association between sleep duration per night and executive function at baseline in women

Sleep duration per night, hours	ELSA (n=5174)		CHARLS（n=5386)		Pooled analysis (n=10 560)			
	LSM of Global Z scores[a]	*P* value	LSM of Global Z scores[a]	*P* value	Pooled LSM of Global Z scores	*P* value	I^2 (%)	*P* value
≤4	-0.19 (-0.30 to -0.07)	.001	-0.17 (-0.26 to -0.08)	<0.001	-0.18 (-0.25 to -0.10)	<0.001	0.0	.79
5	-0.10 (-0.19 to -0.01)	.04	-0.04 (-0.13 to 0.05)	.41	-0.07 (-0.14 to 0.00)	0.04	0.0	.39
6	-0.05 (-0.12 to 0.03)	.21	0.00 (-0.08 to 0.08)	.98	-0.03 (-0.08 to 0.03)	0.34	0.0	.42
7	Reference	/	Reference	/	Reference	/	/	/
8	-0.08 (-0.15 to -0.02)	.01	-0.08 (-0.17 to 0.00)	.05	-0.08 (-0.14 to -0.03)	0.001	0.0	1.00
9	-0.15 (-0.27 to -0.03)	.01	-0.29 (-0.44 to -0.14)	<0.001	-0.21 (-0.34 to -0.08)	0.002	47.1	.17
≥10	-0.39 (-0.58 to -0.20)	<0.001	-0.34 (-0.49 to -0.20)	<0.001	-0.36 (-0.47 to -0.24)	<0.001	0.0	.73

Abbreviations: ELSA, the English Longitudinal Study of Ageing; CHARLS, the China Health Retirement Longitudinal Study; LSM, least-squares means.

[a] Data are presented as LSM and 95% confidence interval after adjusting for age, education, body mass index, the Center for Epidemiologic Studies Depression Scale score, systolic blood pressure, cohabitation status, current smoking, alcohol consumption, diabetes, coronary heart disease, stroke, cancer, chronic lung disease, and asthma.

© 2020 Ma Y et al. *JAMA Network Open*.

eTable 16. Association between sleep duration per night and orientation function at baseline in women

Sleep duration per night, hours	ELSA (n=5174)			CHARLS（n=5386)			Pooled analysis (n=10 560)			
	LSM of orientation Z scores[a]	*P* value		LSM of orientation Z scores[a]	*P* value		Pooled LSM of orientation Z scores	*P* value	I^2 (%)	*P* value
≤4	-0.06 (-0.19 to 0.06)	.31		-0.08 (-0.18 to 0.01)	.08		-0.08 (-0.15 to 0.00)	0.04	0.0	.80
5	-0.03 (-0.13 to 0.07)	.52		-0.01 (-0.11 to 0.08)	.79		-0.02 (-0.09 to 0.05)	0.53	0.0	.78
6	0.00 (-0.08 to 0.07)	.93		-0.01 (-0.09 to 0.08)	.87		-0.01 (-0.06 to 0.05)	0.86	0.0	.95
7	Reference	/		Reference	/		Reference	/	/	/
8	-0.04 (-0.11 to 0.03)	.23		-0.04 (-0.12 to 0.05)	.40		-0.04 (-0.09 to 0.01)	0.14	0.0	.89
9	-0.12 (-0.24 to 0.01)	.07		-0.23 (-0.39 to -0.08)	.003		-0.17 (-0.28 to -0.05)	0.004	23.7	.25
≥10	-0.68 (-0.89 to -0.48)	<0.001		-0.29 (-0.44 to -0.15)	<0.001		-0.48 (-0.86 to -0.10)	0.01	89.3	.002

Abbreviations: ELSA, the English Longitudinal Study of Ageing; CHARLS, the China Health Retirement Longitudinal Study; LSM, least-squares means.

[a] Data are presented as LSM and 95% confidence interval after adjusting for age, education, body mass index, the Center for Epidemiologic Studies Depression Scale score, systolic blood pressure, cohabitation status, current smoking, alcohol consumption, diabetes, coronary heart disease, stroke, cancer, chronic lung disease, and asthma.

© 2020 Ma Y et al. *JAMA Network Open*.

eTable 17. Association between sleep duration and global cognitive decline (SD/year) during follow-up in men

Sleep duration per night, hours	ELSA (n=4080)			CHARLS（n=5425)			Pooled analysis (n=9505)			
	**β (95% CI)**	***P* value**[a]		**β (95% CI)**	***P* value**[a]		**Pooled β (95% CI)**	***P* value**	I^2 (%)	***P* value**
≤4	-0.034 (-0.064 to -0.003)	.03		-0.029 (-0.059 to 0.001)	.06		-0.031 (-0.053 to -0.010)	0.004	0.0	.83
5	-0.010 (-0.032 to 0.012)	.37		-0.010 (-0.040 to 0.020)	.51		-0.010 (-0.028 to 0.008)	0.26	0.0	.99
6	0.001 (-0.013 to 0.016)	.88		-0.004 (-0.030 to 0.022)	.77		0.000 (-0.013 to 0.013)	0.99	0.0	.74
7	Reference	/		Reference	/		Reference	/	/	/
8	0.001 (-0.012 to 0.014)	.86		-0.004 (-0.029 to 0.021)	.76		0.000 (-0.012 to 0.012)	0.99	0.0	.72
9	-0.011 (-0.037 to 0.016)	.42		-0.005 (-0.052 to 0.041)	.82		-0.009 (-0.032 to 0.013)	0.42	0.0	.84
≥10	-0.039 (-0.085 to 0.007)	.10		-0.050 (-0.095 to -0.005)	.03		-0.045 (-0.077 to -0.013)	0.006	0.0	.74

Abbreviations: ELSA, the English Longitudinal Study of Ageing; CHARLS, the China Health Retirement Longitudinal Study.

[a] After adjusting for age, education, body mass index, the Center for Epidemiologic Studies Depression Scale score, systolic blood pressure, cohabitation status, current smoking, alcohol consumption, diabetes, coronary heart disease, stroke, cancer, chronic lung disease, and asthma.

© 2020 Ma Y et al. *JAMA Network Open.*

eTable 18. Association between sleep duration and memory decline (SD/year) during follow-up in men

Sleep duration per night, hours	ELSA (n=4080)			CHARLS（n=5425)			Pooled analysis (n=9505)			
	β (95% CI)	*P* value[a]		β (95% CI)	*P* value[a]		Pooled β (95% CI)	*P* value	I^2 (%)	*P* value
≤4	-0.013 (-0.036 to 0.010)	.26		-0.037 (-0.071 to -0.002)	.04		-0.021 (-0.043 to 0.001)	0.06	20.6	.26
5	-0.003 (-0.019 to 0.014)	.76		-0.043 (-0.077 to -0.009)	.01		-0.020 (-0.059 to 0.019)	0.32	77.3	.04
6	0.000 (-0.011 to 0.011)	.94		-0.026 (-0.056 to 0.003)	.08		-0.009 (-0.035 to 0.016)	0.47	64.3	.09
7	Reference	/		Reference	/		Reference	/	/	/
8	0.004 (-0.006 to 0.014)	.41		-0.017 (-0.046 to 0.011)	.24		-0.002 (-0.021 to 0.017)	0.82	47.9	.17
9	-0.007 (-0.027 to 0.013)	.48		-0.062 (-0.115 to -0.009)	.02		-0.029 (-0.082 to 0.024)	0.28	72.6	.06
≥10	-0.023 (-0.057 to 0.012)	.19		-0.027 (-0.079 to 0.025)	.30		-0.024 (-0.053 to 0.004)	0.10	0.0	.89

Abbreviations: ELSA, the English Longitudinal Study of Ageing; CHARLS, the China Health Retirement Longitudinal Study.

[a] After adjusting for age, education, body mass index, the Center for Epidemiologic Studies Depression Scale score, systolic blood pressure, cohabitation status, current smoking, alcohol consumption, diabetes, coronary heart disease, stroke, cancer, chronic lung disease, and asthma.

© 2020 Ma Y et al. *JAMA Network Open.*

eTable 19. Association between sleep duration and executive decline (SD/year) during follow-up in men

Sleep duration per night, hours	ELSA (n=4080)		CHARLS（n=5425)		Pooled analysis (n=9505)			
	β (95% CI)	*P* value[a]	β (95% CI)	*P* value[a]	Pooled β (95% CI)	*P* value	I^2 (%)	*P* value
≤4	-0.015 (-0.040 to 0.010)	.24	0.025 (0.002 to 0.048)	.03	0.005 (-0.034 to 0.044)	0.79	81.1	.02
5	-0.017 (-0.035 to 0.000)	.05	0.003 (-0.020 to 0.025)	.81	-0.009 (-0.028 to 0.011)	0.39	48.0	.17
6	0.000 (-0.012 to 0.012)	.99	0.009 (-0.010 to 0.028)	.36	0.003 (-0.008 to 0.013)	0.63	0.0	.44
7	Reference	/	Reference	/	Reference	/	/	/
8	-0.008 (-0.019 to 0.002)	.12	0.007 (-0.012 to 0.026)	.46	-0.003 (-0.017 to 0.012)	0.72	48.8	.16
9	-0.017 (-0.039 to 0.004)	.11	0.039 (0.003 to 0.074)	.03	0.009 (-0.046 to 0.063)	0.75	85.9	.008
≥10	-0.035 (-0.072 to 0.003)	.07	-0.003 (-0.037 to 0.032)	.89	-0.018 (-0.049 to 0.014)	0.27	35.4	.21

Abbreviations: ELSA, the English Longitudinal Study of Ageing; CHARLS, the China Health Retirement Longitudinal Study.

[a] After adjusting for age, education, body mass index, the Center for Epidemiologic Studies Depression Scale score, systolic blood pressure, cohabitation status, current smoking, alcohol consumption, diabetes, coronary heart disease, stroke, cancer, chronic lung disease, and asthma.

© 2020 Ma Y et al. *JAMA Network Open.*

eTable 20. Association between sleep duration and orientation decline (SD/year) during follow-up in men

Sleep duration per night, hours	ELSA (n=4080)			CHARLS（n=5425)			Pooled analysis (n=9505)			
	β (95% CI)[a]	*P* value		β (95% CI)[a]	*P* value		Pooled β (95% CI)	*P* value	I^2 (%)	*P* value
≤4	-0.051 (-0.090 to -0.012)	.01		-0.032 (-0.061 to -0.002)	.04		-0.039 (-0.062 to -0.015)	0.001	0.0	.45
5	-0.008 (-0.035 to 0.020)	.58		0.002 (-0.027 to 0.032)	.88		-0.003 (-0.023 to 0.017)	0.77	0.0	.63
6	0.002 (-0.017 to 0.020)	.86		0.000 (-0.026 to 0.025)	.97		0.001 (-0.014 to 0.016)	0.91	0.0	.89
7	Reference	/		Reference	/		Reference	/	/	/
8	0.009 (-0.008 to 0.026)	.30		-0.009 (-0.034 to 0.016)	.49		0.003 (-0.014 to 0.019)	0.76	25.0	.25
9	0.007 (-0.027 to 0.041)	.69		0.012 (-0.034 to 0.058)	.61		0.009 (-0.018 to 0.036)	0.53	0.0	.86
≥10	0.001 (-0.057 to 0.060)	.96		-0.043 (-0.087 to 0.002)	.06		-0.025 (-0.067 to 0.017)	0.25	28.0	.24

Abbreviations: ELSA, the English Longitudinal Study of Ageing; CHARLS, the China Health Retirement Longitudinal Study.

[a] After adjusting for age, education, body mass index, the Center for Epidemiologic Studies Depression Scale score, systolic blood pressure, cohabitation status, current smoking, alcohol consumption, diabetes, coronary heart disease, stroke, cancer, chronic lung disease, and asthma.

© 2020 Ma Y et al. *JAMA Network Open.*

eTable 21. Association between sleep duration and global cognitive decline (SD/year) during follow-up in women

Sleep duration per night, hours	ELSA (n=5174)			CHARLS（n=5386)			Pooled analysis (n=10 560)			
	β (95% CI)	*P* value[a]		β (95% CI)	*P* value[a]		Pooled β (95% CI)	*P* value	I^2 (%)	*P* value
≤4	-0.016 (-0.038 to 0.005)	.14		-0.020 (-0.048 to 0.009)	.17		-0.017 (-0.035 to 0.000)	0.05	0.0	.85
5	-0.002 (-0.020 to 0.015)	.79		-0.004 (-0.034 to 0.026)	.78		-0.003 (-0.018 to 0.012)	0.71	0.0	.91
6	-0.010 (-0.023 to 0.003)	.15		-0.003 (-0.030 to 0.023)	.82		-0.008 (-0.020 to 0.003)	0.16	0.0	.66
7	Reference	/		Reference	/		Reference	/	/	/
8	-0.007 (-0.020 to 0.005)	.23		0.001 (-0.025 to 0.028)	.91		-0.006 (-0.017 to 0.005)	0.30	0.0	.55
9	-0.033 (-0.055 to -0.010)	.004		0.024 (-0.024 to 0.073)	.33		-0.009 (-0.064 to 0.047)	0.76	77.1	.04
≥10	-0.025 (-0.063 to 0.013)	.19		-0.022 (-0.068 to 0.024)	.36		-0.024 (-0.053 to 0.006)	0.11	0.0	.91

Abbreviations: ELSA, the English Longitudinal Study of Ageing; CHARLS, the China Health Retirement Longitudinal Study.

[a] After adjusting for age, education, body mass index, the Center for Epidemiologic Studies Depression Scale score, systolic blood pressure, cohabitation status, current smoking, alcohol consumption, diabetes, coronary heart disease, stroke, cancer, chronic lung disease, and asthma.

© 2020 Ma Y et al. *JAMA Network Open.*

eTable 22. Association between sleep duration and memory decline (SD/year) during follow-up in women

Sleep duration per night, hours	ELSA (n=5174)			CHARLS（n=5386)			Pooled analysis (n=10 560)			
	β (95% CI)	*P* value[a]		β (95% CI)	*P* value[a]		Pooled β (95% CI)	*P* value	I^2 (%)	*P* value
≤4	-0.011 (-0.027 to 0.006)	.20		-0.035 (-0.067 to -0.003)	.03		-0.019 (-0.041 to 0.003)	0.10	41.3	.19
5	0.000 (-0.013 to 0.013)	.97		-0.008 (-0.042 to 0.026)	.64		-0.001 (-0.013 to 0.011)	0.90	0.0	.65
6	-0.010 (-0.021 to 0.000)	.04		-0.011 (-0.041 to 0.019)	.47		-0.010 (-0.020 to -0.001)	0.03	0.0	.98
7	Reference	/		Reference	/		Reference	/	/	/
8	-0.001 (-0.010 to 0.008)	.84		-0.006 (-0.035 to 0.024)	.71		-0.001 (-0.010 to 0.008)	0.76	0.0	.77
9	-0.016 (-0.033 to 0.001)	.06		-0.005 (-0.059 to 0.049)	.86		-0.015 (-0.031 to 0.001)	0.07	0.0	.70
≥10	-0.003 (-0.032 to 0.026)	.83		-0.056 (-0.108 to -0.004)	.03		-0.025 (-0.076 to 0.026)	0.34	66.9	.08

Abbreviations: ELSA, the English Longitudinal Study of Ageing; CHARLS, the China Health Retirement Longitudinal Study.

[a] After adjusting for age, education, body mass index, the Center for Epidemiologic Studies Depression Scale score, systolic blood pressure, cohabitation status, current smoking, alcohol consumption, diabetes, coronary heart disease, stroke, cancer, chronic lung disease, and asthma.

© 2020 Ma Y et al. *JAMA Network Open.*

eTable 23. Association between sleep duration and executive decline (SD/year) during follow-up in women

Sleep duration per night, hours	ELSA (n=5174)			CHARLS（n=5386)			Pooled analysis (n=10 560)			
	β (95% CI)	*P* value[a]		β (95% CI)	*P* value[a]		Pooled β (95% CI)	*P* value[a]	I^2 (%)	*P* value
≤4	-0.025 (-0.041 to -0.008)	.004		0.018 (-0.006 to 0.042)	.13		-0.004 (-0.046 to 0.038)	0.85	88.0	.004
5	-0.008 (-0.021 to 0.006)	.25		-0.004 (-0.029 to 0.020)	.72		-0.007 (-0.019 to 0.005)	0.24	0.0	.81
6	-0.010 (-0.020 to 0.001)	.07		0.015 (-0.007 to 0.037)	.18		0.001 (-0.023 to 0.024)	0.95	74.8	.05
7	Reference	/		Reference	/		Reference	/	/	/
8	-0.009 (-0.019 to 0.000)	.06		0.013 (-0.009 to 0.034)	.25		-0.001 (-0.022 to 0.021)	0.96	69.8	.07
9	-0.011 (-0.029 to 0.006)	.20		0.016 (-0.024 to 0.056)	.44		-0.004 (-0.027 to 0.019)	0.74	31.9	.23
≥10	0.004 (-0.026 to 0.033)	.80		0.025 (-0.014 to 0.064)	.21		0.012 (-0.012 to 0.035)	0.33	0.0	.40

Abbreviations: ELSA, the English Longitudinal Study of Ageing; CHARLS, the China Health Retirement Longitudinal Study.

[a] After adjusting for age, education, body mass index, the Center for Epidemiologic Studies Depression Scale score, systolic blood pressure, cohabitation status, current smoking, alcohol consumption, diabetes, coronary heart disease, stroke, cancer, chronic lung disease, and asthma.

© 2020 Ma Y et al. *JAMA Network Open.*

eTable 24. Association between sleep duration and orientation decline (SD/year) during follow-up in women

Sleep duration per night, hours	ELSA (n=5174)			CHARLS（n=5386)			Pooled analysis (n=10 560)			
	β (95% CI)	*P* value[a]		β (95% CI)	*P* value[a]		Pooled β (95% CI)	*P* value[a]	I^2 (%)	*P* value
≤4	-0.006 (-0.033 to 0.021)	.65		-0.008 (-0.037 to 0.021)	.59		-0.007 (-0.027 to 0.013)	0.49	0.0	.93
5	0.002 (-0.020 to 0.024)	.86		0.012 (-0.019 to 0.043)	.45		0.005 (-0.012 to 0.023)	0.56	0.0	.60
6	-0.005 (-0.022 to 0.011)	.54		0.010 (-0.017 to 0.038)	.46		-0.001 (-0.015 to 0.013)	0.89	0.0	.34
7	Reference	/		Reference	/		Reference	/	/	/
8	-0.008 (-0.024 to 0.007)	.30		0.007 (-0.021 to 0.034)	.63		-0.005 (-0.018 to 0.009)	0.50	0.0	.35
9	-0.032 (-0.060 to -0.004)	.03		0.038 (-0.012 to 0.088)	.13		0.000 (-0.068 to 0.069)	1.00	82.6	.02
≥10	-0.041 (-0.088 to 0.006)	.09		0.010 (-0.038 to 0.058)	.68		-0.016 (-0.066 to 0.035)	0.54	55.3	.14

Abbreviations: ELSA, the English Longitudinal Study of Ageing; CHARLS, the China Health Retirement Longitudinal Study.

[a] After adjusting for age, education, body mass index, the Center for Epidemiologic Studies Depression Scale score, systolic blood pressure, cohabitation status, current smoking, alcohol consumption, diabetes, coronary heart disease, stroke, cancer, chronic lung disease, and asthma.

© 2020 Ma Y et al. *JAMA Network Open.*

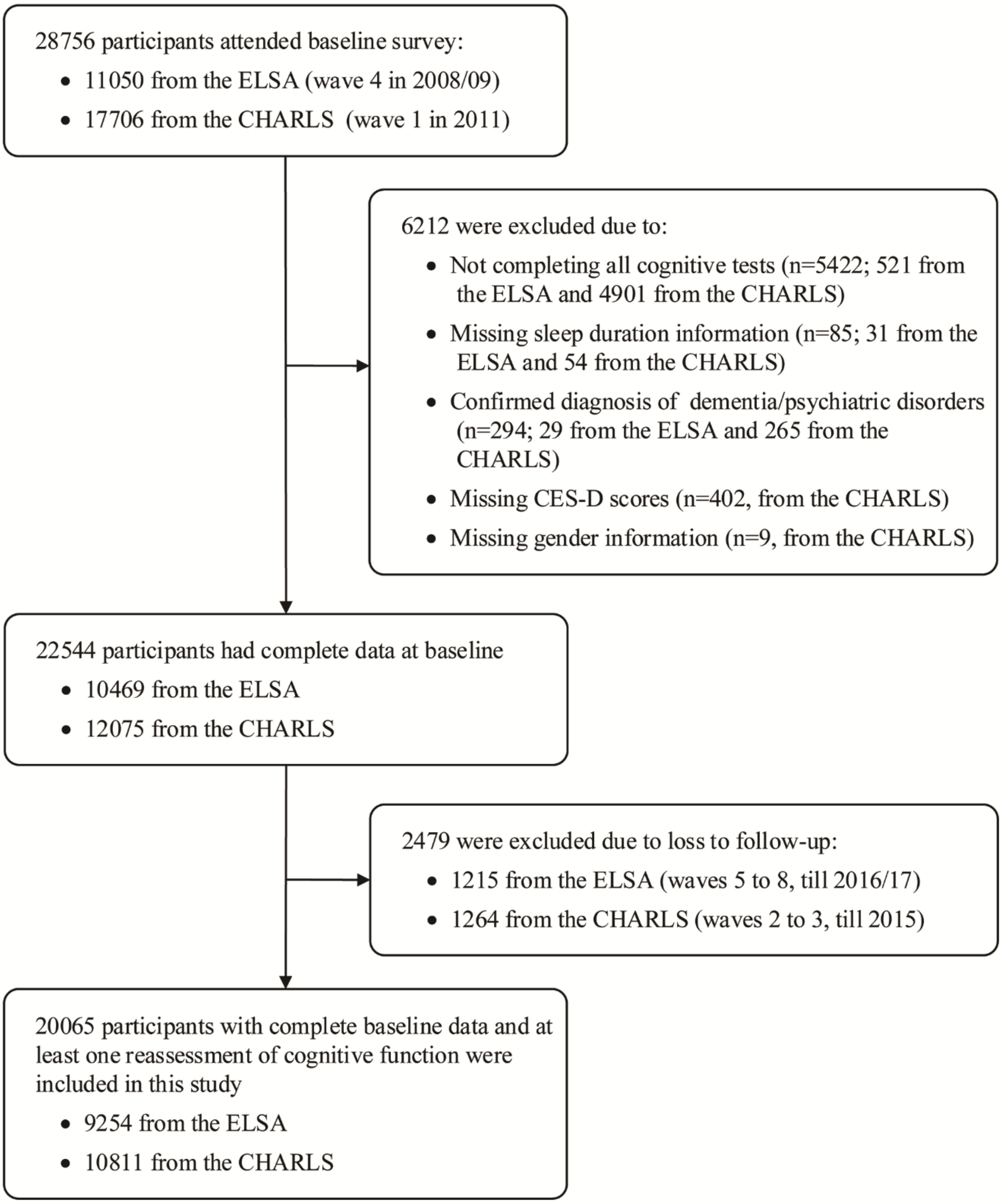

eFigure 1. Flow chart of participant selection for this study.

© 2020 Ma Y et al. *JAMA Network Open*.

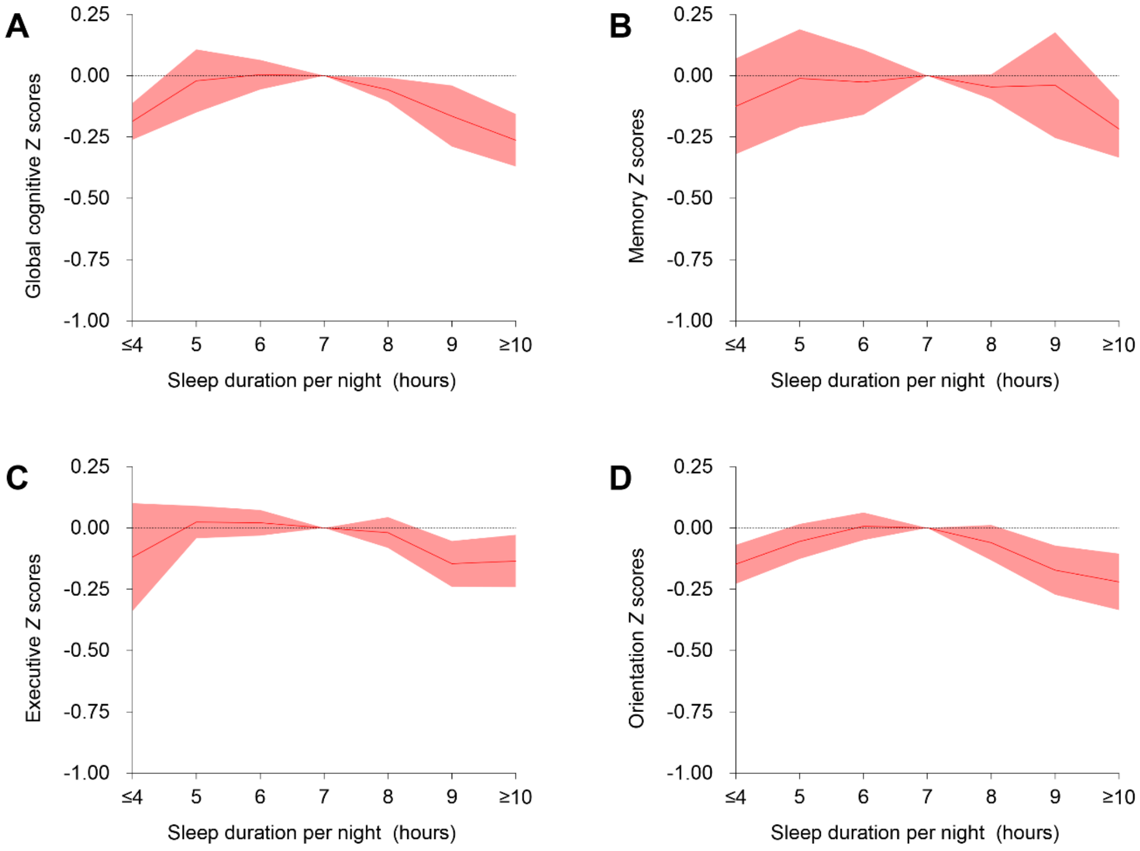

eFigure 2. Cross-sectional associations between sleep duration per night and cognitive functions at baseline in men: pooled results of two cohorts

Participants who had a sleep duration of 7 hours per night as the reference group. Solid lines represent adjusted least-squares means after adjusting for age, education, body mass index, the Center for Epidemiologic Studies Depression Scale score, systolic blood pressure, cohabitation status, current smoking, alcohol consumption, diabetes, coronary heart disease, stroke, cancer, chronic lung disease, and asthma. The shadows represent the 95% CIs.

© 2020 Ma Y et al. *JAMA Network Open.*

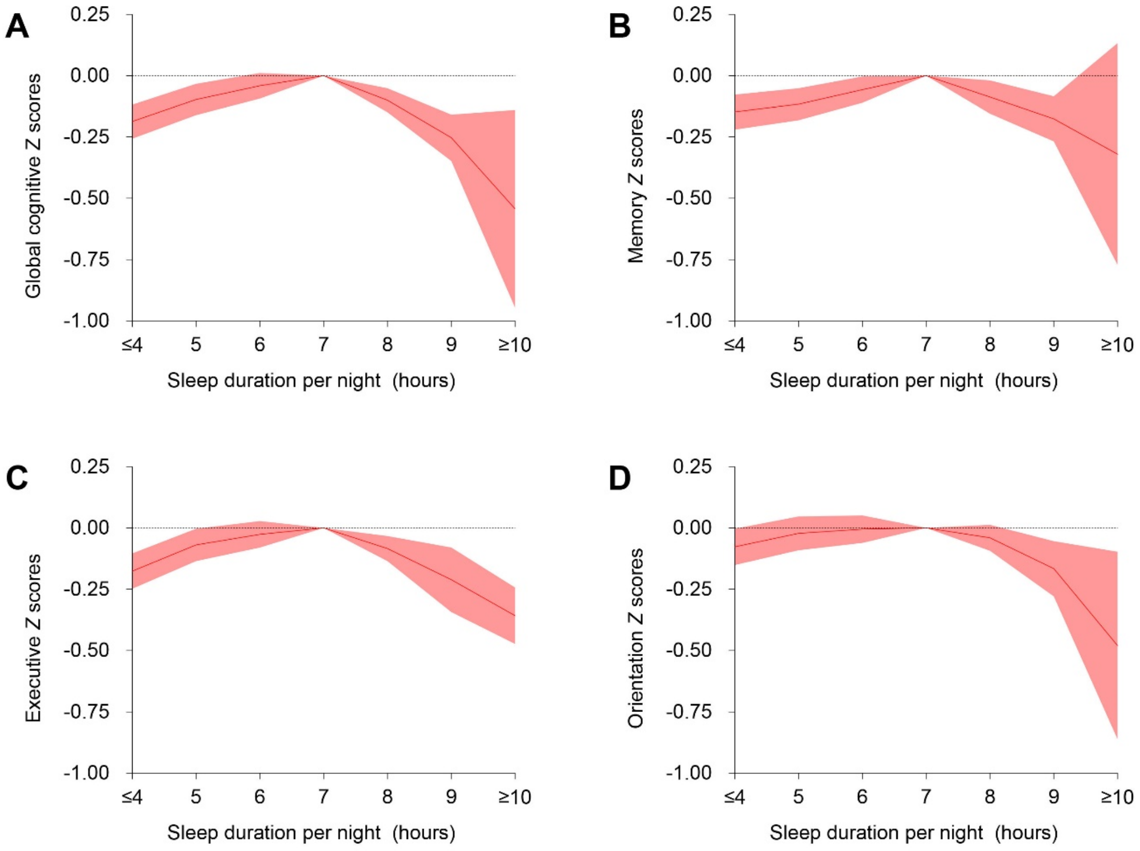

eFigure 3. Cross-sectional associations between sleep duration per night and cognitive functions at baseline in women: pooled results of two cohorts

Participants who had a sleep duration of 7 hours per night as the reference group. Solid lines represent adjusted least-squares means after adjusting for age, education, body mass index, the Center for Epidemiologic Studies Depression Scale score, systolic blood pressure, cohabitation status, current smoking, alcohol consumption, diabetes, coronary heart disease, stroke, cancer, chronic lung disease, and asthma. The shadows represent the 95% CIs.

© 2020 Ma Y et al. *JAMA Network Open.*

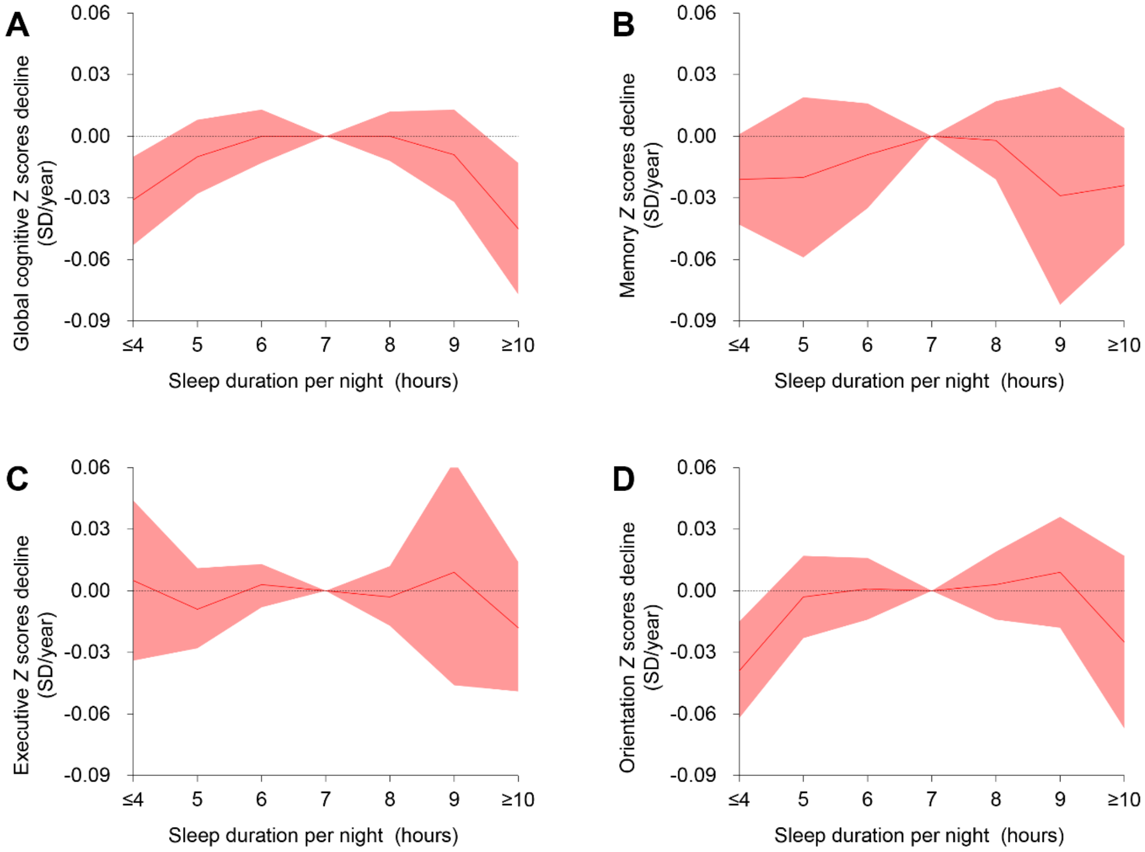

eFigure 4. Longitudinal associations between sleep duration per night and cognitive declines during follow-up in men: pooled results of two cohorts

Participants who had a sleep duration of 7 hours per night as the reference group. Solid lines represent adjusted mean differences after adjusting for age, education, body mass index, the Center for Epidemiologic Studies Depression Scale score, systolic blood pressure, cohabitation status, current smoking, alcohol consumption, diabetes, coronary heart disease, stroke, cancer, chronic lung disease, and asthma. The shadows represent the 95% CIs.

© 2020 Ma Y et al. *JAMA Network Open*.

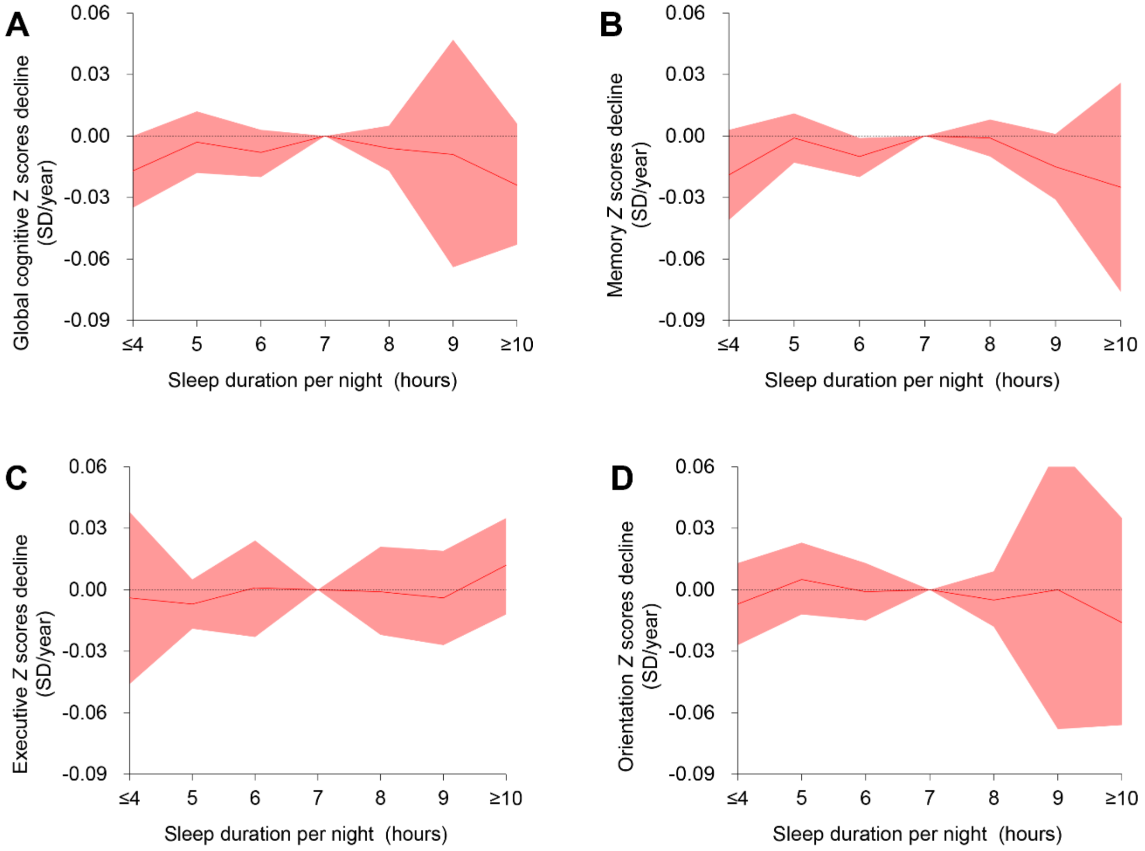

eFigure 5. Longitudinal associations between sleep duration per night and cognitive declines during follow-up in women: pooled results of two cohorts

Participants who had a sleep duration of 7 hours per night as the reference group. Solid lines represent adjusted mean differences after adjusting for age, education, body mass index, the Center for Epidemiologic Studies Depression Scale score, systolic blood pressure, cohabitation status, current smoking, alcohol consumption, diabetes, coronary heart disease, stroke, cancer, chronic lung disease, and asthma. The shadows represent the 95% CIs.

© 2020 Ma Y et al. *JAMA Network Open.*
